# Incivility Diminishes Interest in What Politicians Have to Say

**DOI:** 10.1177/19485506221136182

**Published:** 2022-11-16

**Authors:** Matthew Feinberg, Jeremy A. Frimer

**Affiliations:** 1 University of Toronto, Ontario, Canada; 2 The University of Winnipeg, Manitoba, Canada

**Keywords:** Incivility, rudeness, interest, following, politics

## Abstract

Incivility is prevalent in society suggesting a potential benefit. Within politics, theorists and strategists often claim incivility grabs attention and stokes interest in what a politician has to say. In contrast, we propose incivility diminishes overall interest in what a politician has to say because people find the incivility morally distasteful. Studies 1a and 1b examined the relationship between uncivil language and followership in the Twitter feeds of Presidents Donald Trump and Joe Biden, finding incivility reduced their following on the platform. In Studies 2–3, we manipulated how uncivil a number of politicians were and found that incivility consistently depressed interest in what they had to say. These effects of incivility are generalized to both political allies and opponents. Observers’ moral disapproval of the incivility mediated the diminished interest, suppressing the attention-grabbing nature of incivility. Altogether, our findings indicate that the public reacts more negatively to political incivility than previously thought.

Incivility is prevalent in society. We often experience, or hear of other’s encounters with, people acting uncivilly. Research has found such behavior imposes substantial costs on the uncivil actor (Carraro & Castelli, 2010; Ng & Detenber, 2005; Tyler & Blader, 2000). Yet, the continuing prevalence of incivility suggests some may view it as a useful means to achieving certain goals, such as getting people to listen to what they have to say. In the present research, we examine this possibility in the domain of politics, where incivility is often commonplace, especially in polarized environments like the United States.

Although scholars have not settled on a single definition of incivility, building on past work (e.g., Massaro & Stryker, 2012; Schilpzand et al., 2016; Sobieraj & Berry, 2011), we define incivility as the use of rude, disrespectful language aimed at insulting or demeaning others. In political contexts, this often manifests as name-calling, derision, and sometimes vulgarity (see Stryker et al., 2016). It is widely theorized that politicians use incivility to draw the public’s attention to what they have to say, which yields a broader audience and political influence (e.g., Berry & Sobieraj, 2014; Brady et al., 2017; Mutz, 2015; Pinkleton & Garramone, 1992). At first glance, this assumption appears viable: Notoriously uncivil politicians often gain media attention and become household names. But does incivility in fact pique interest in hearing more of what the uncivil actor has to say?

In the present research, we challenge existing notions that incivility results in increased interest. We argue incivility *reduces* observers’ interest in hearing what a politician has to say because people’s moral disapproval of incivility outweighs its attention-grabbing nature. We explore this possibility across four studies—two field and two experimental—while also examining the possibility that party affiliation moderates the effect.

## Ambivalent Reactions to Incivility

Incivility tends to evoke ambivalent reactions. People are attracted to incivility because it grabs their attention. Onlookers find incivility entertaining, arousing, and memorable (Brader, 2005; Kosmides & Theocharis, 2020; Mutz & Reeves, 2005). For instance, Mutz and Reeves (2005) presented participants with either civil or uncivil political debates and found those watching the uncivil debate were more entertained and exhibited greater physiological arousal. Moreover, incivility inspires people to share uncivil content with others (e.g., Feinberg et al., 2012; Muddiman & Stroud, 2017). Brady et al. (2017) found uncivil tweets received many more “retweets” than civil ones. Yet, uncivil behavior is also met with moral disapproval. Observing incivility evokes anger and outrage toward uncivil content and its messenger (Coe et al., 2014; Gervais, 2018), and observers distance themselves from the uncivil. For example, Feinberg et al. (2020) found observers felt psychologically distant from and less supportive of protesters who behaved uncivilly.

**Figure 1:**
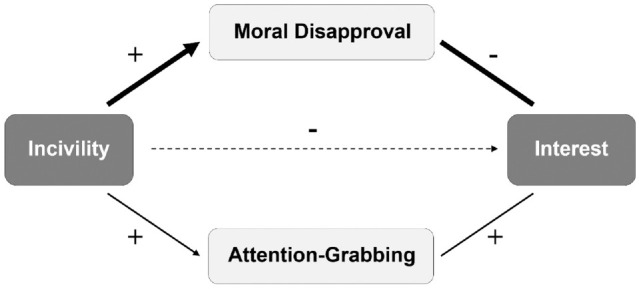
Theoretical Model of How Responses of Incivility Affect Interest. *Note.* Bolded lines indicate the stronger pathway.

This analysis indicates that incivility evokes countervailing responses—one drawing people toward the source (attention-grabbing) and one repelling them away (moral disapproval). How individuals balance these responses will determine whether they are, in the end, interested in hearing what an uncivil politician has to say. Importantly, moral judgments take precedence and supersede other social judgments (Brambilla et al., 2021). In fact, studies find being moral is the most desirable characteristic a person can possess (Cottrell et al., 2007). Drawing on this “primacy of morality” (Brambilla et al., 2011), we propose a model (Figure 1) whereby the loss of interest due to incivility evoking moral disapproval *outweighs* the gains of incivility being attention-grabbing. The result is decreased interest; individuals will not want to hear more of what an uncivil politician has to say.

Of note, our theorizing extends work on incivility’s role in affecting a politician’s reputation. Frimer and Skitka (2018) found politicians’ incivility resulted in individuals judging them as less warm and likable. Although informative, this past work is agnostic about whether the negative reputation uncivil politicians incur positively or negatively impacts levels of interest. Indeed, it may be that being disliked results in greater interest in what a politician has to say in the same way individuals find villains and antagonists intriguing (e.g., The Joker, Darth Vader; Vice, 2005). An uncivil, attention-grabbing style might provide a platform for a politician to be heard even if disliked.

Finally, incivility might affect interest differently between and within political groups. When a politician makes uncivil comments, individuals who identify with the same political party (“co-partisans”) as the uncivil actor might not morally disapprove and therefore not experience diminished interest. We might even expect incivility to produce greater interest. Related work on this topic provides mixed insights, with some suggesting party affiliation will moderate how individuals respond to uncivil politicians (Anderson et al., 2014; Druckman et al., 2018; Lau & Pomper, 2001), while other work suggests little or no influence (Feinberg et al., 2020; Frimer & Skitka, 2018). Although our primary focus was on the main effects of incivility and their underlying mechanisms, in the present research we also explore the possibility of the political party as a moderator.

## The Present Research

The goal of the present research was to test whether incivility decreases interest in what a politician has to say. Studies 1a and b were longitudinal analyses of the Twitter feeds of Presidents Donald Trump and Joe Biden testing whether times when their Twitter feeds were particularly uncivil predicted reductions in new followers their Twitter accounts accrued. These studies allowed us to test our theorizing in a real-world, ecologically valid context. Studies 2 and 3 experimentally manipulated whether Democratic and Republican participants observed civil or uncivil messages from Democratic and Republican politicians and examined interest in consuming more information from the source, allowing us to explore whether the interest-depressing effect of incivility generalized across political party identification. Study 3 also measured how much participants found the politician’s behavior to be attention-grabbing and worthy of moral disapproval, which allowed us to test our model for why incivility results in decreased interest in a politician. Specifically, we expected the interest-depressing effect of morally disapproving of incivility would suppress the interest-raising effect of finding it attention-grabbing, resulting in an overall reduction in interest.

## Study 1a

Study 1 examined Donald Trump’s Twitter feed and associated changes in the number of people following his Twitter account. Although Donald Trump’s candidacy and presidency were often characterized as uncivil, he won the 2016 Presidential election and enjoyed widespread approval among Republicans throughout his presidency. This could mean his incivility helped him gain a following of interested observers. However, it is also possible Trump won the presidency and maintained support *in spite* of his incivility. To test this, we examined whether his followership on Twitter grew faster or slower when his Twitter feed was particularly uncivil.

### Method

#### Procedure

We assessed the temporal relationship between levels of incivility in the tweets issued by former President Trump and the number of followers his Twitter account had the following day, suggestive of a causal effect of incivility on interest in hearing more of what he had to say.

#### Uncivil Tweets

The corpus was from the Trump Twitter Archive (https://www.thetrumparchive.com/faq); we included all tweets by Donald Trump beginning on June 8, 2015, when he began his run for president, and ending on January 8, 2021, when Twitter permanently suspended the account. During this time, Trump issued 32,882 tweets (*M* = 16 daily).

Each tweet was coded for its level of incivility on a scale from 0 to 1 by Google’s PerspectiveAPI. PerspectiveAPI is an artificial intelligence classifier trained to detect rude or disrespectful language using machine-learning techniques (https://cran.r-project.org/web/packages/peRspective/peRspective.pdf). Frimer, Aujla, FEinberg, Skitka, Aquino, Eichstaedt, & Willer (2022) demonstrated the validity of PerspectiveAPI on political tweets by showing that it converges with human ratings of incivility, *r* = .63. The unit of analysis was the day; we averaged the incivility scores from all tweets issued on a given day, *M* = .20, *SD* = .07, range: .02–.66.

#### Twitter Followers

Following someone on Twitter indicates a desire to have all future posts from the account appear in one’s Twitter feed. Therefore, the following is a behavioral expression of wanting to hear more from a person.

The data set, collected from https://factba.se/topic/twitter-stats, included the number of accounts following the Twitter feed over the period under study. Data were available 99% of the days. Followers to Trump’s Twitter feed rose from ∼3 million to ∼89 million.

### Results

**Figure 2:**
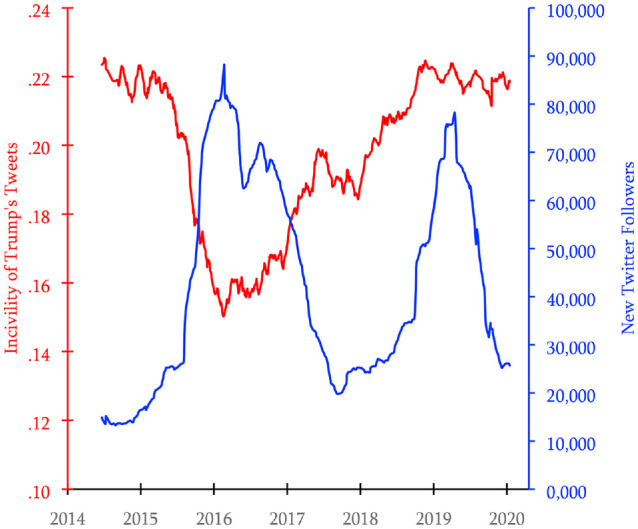
Levels of Incivility and Numbers of New Followers of President Donald Trump’s Twitter Handle Between Mid-2015 and Early 2021 *Note.* Lines represent 200-day moving averages (Study 1a).

All data are available at https://osf.io/79wkc/?view_only=1f785591e5f44556b0dfae4dd05b6b46. Figure 2 displays trends in incivility from Donald Trump’s Twitter feed and the number of followers gained each day and illustrates the inverse relationship between incivility and follower gains. In 2015–2016, Trump’s tweets were relatively uncivil and he gained relatively few followers. The first two years of his presidency were marked by relatively low incivility and large gains in the number of followers. Incivility rose between 2017 and 2019 and follower gains were relatively modest. From 2019 to 2021, incivility remained elevated and follower gains began high but then declined rapidly in 2020.

To assess the causal relationship between incivility and followers lost, we conducted a Granger causality test. Granger causality tests (Granger, 1969) are a common statistical method used in longitudinal (time series) data that permit causal inferences by testing whether data from earlier time points predict data from later time points while controlling for an autocorrelational effect (Equation 1).

(1) $$\mathrm{Follower}s_{\mathrm{tomorrow}}=a\;\mathrm{Follower}s_{\mathrm{today}}+b\;\mathrm{Incivilit}y_{\mathrm{today}}+e.$$

The analysis indicated incivility significantly predicted lower followership the next day, *B* = −61,962, 95%confidence interval [CI]: [−94,175, −29,750], *p* < .001, meaning incivility “Granger-caused” a decrease in Trump gaining new followers. To interpret this result, we compared the average number of new followers added per day after days in which Trump’s tweets were very civil (*model-implied incivility* = 0) with new followers added after days in which Trump’s tweets were very uncivil (*model-implied incivility* = .7), finding his account received ∼43,000 new followers after days when his Tweet’s were very civil, but only ∼16,000 new followers after days when his tweets were very uncivil. Furthermore, to estimate the total effect of Trump’s incivility on followership, we compared Trump’s incivility in any given day with a realistically low level of incivility he could have displayed (President Obama’s incivility was ∼.13; Frimer et al., 2022). This comparison allowed us to calculate that Trump’s incivility cost him 6,354,137 new followers (see Online Supplementary Materials for analyses relating to potential curvilinearity and thresholds where incivility affects followership).

## Study 1b

To assess whether the real-world effects of incivility on followership replicate with another politician, we examined whether incivility levels in President Joe Biden’s Twitter feed also predicted a subsequent decline in followership.

### Method

#### Procedure

The procedure was the same as in Study 1a. The Biden Twitter corpus was 7048 tweets acquired from https://www.kaggle.com/rohanrao/joe-biden-tweets for tweets from April 9, 2012 (Biden’s first tweet) until March 16, 2020, and through the Twitter API for tweets from March 16, 2020, until June 23, 2021 (when data analyses on this study began). Although the Biden corpus spanned 3,362 days, most tweeting took place since Biden announced his run for president on April 25, 2020. In the 2,572 prior to the announcement, Biden tweeted on only 20% of days. He tweeted 98% of days since the announcement. We therefore included data from the 791 days between April 25, 2020, and June 23, 2021. The sample included 5,697 tweets (*M* = 7 daily). The unit of analysis was the day; we averaged the incivility scores from all tweets issued on a given day, *M* = .15, standard deviation (*SD*) = .07, range: .01–.54. Follower data were available for 99% of days. Followers to his Twitter feed rose from ∼5 million to ∼32 million.

### Results

**Figure 3:**
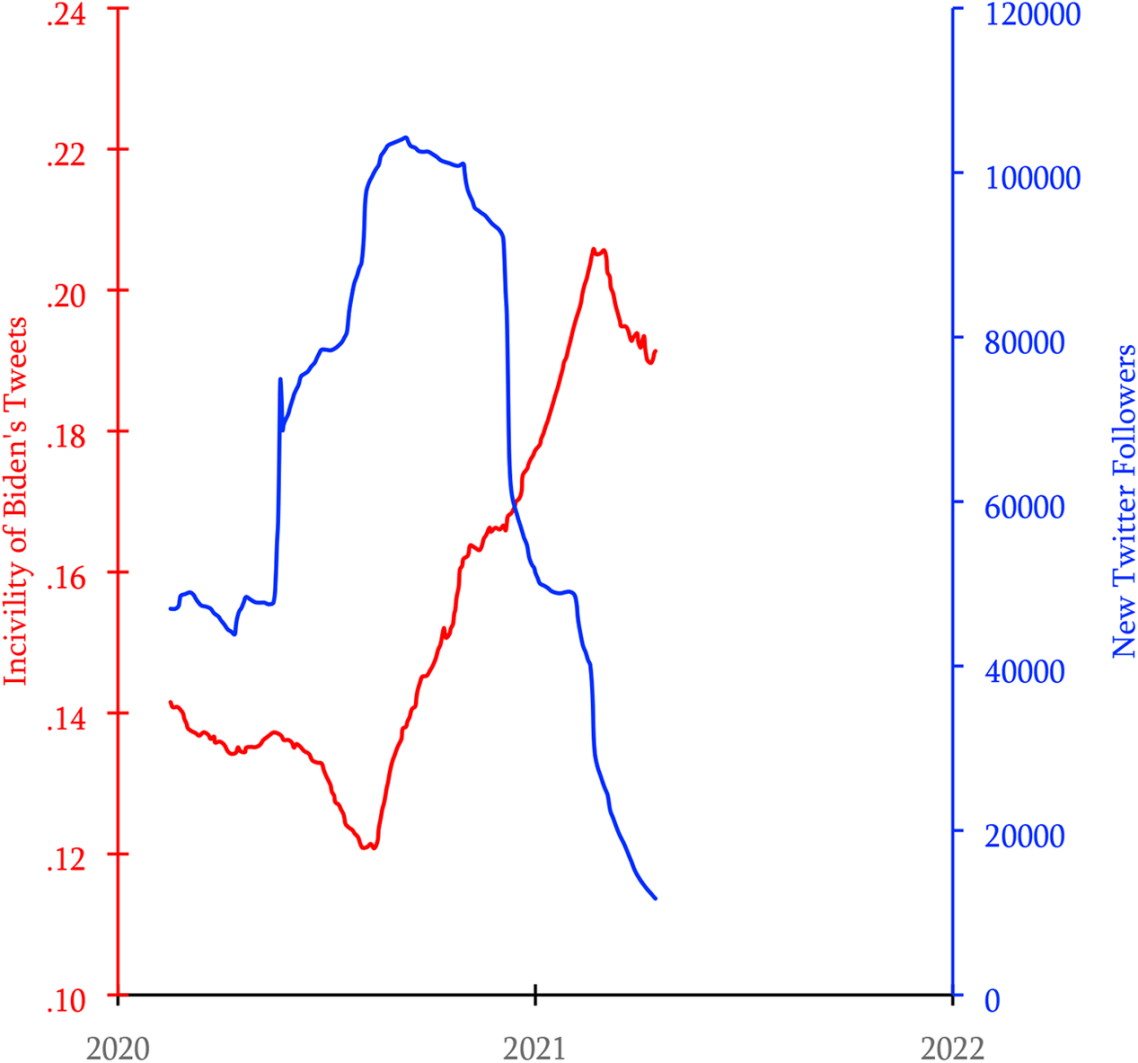
Levels of Incivility and Numbers of New Followers of President Joe Biden’s Twitter Handle Between Early 2020 and Early 2021 *Note.* Lines represent 200-day moving averages (Study 1b).

As in Study 1a, incivility predicted less followership the next day, *B* = −248,798, 95%CI [−388,585, −109,001], *p* < .001, meaning that incivility “Granger-caused” a decrease in Biden gaining new followers. Figure 3 shows the inverse relationship between incivility and new follower counts in the Twitter feed of President Biden.

We interpret this result analogously to how we interpreted the Trump results and found that finding Biden’s account received ∼45,000 new followers after days when his Tweets were very civil, but only ∼11,000 new followers after days when his tweets were very uncivil (incivility = .54). In total, Biden’s incivility cost him 588,156 new followers.

### Discussion

Studies 1a and 1b found Donald Trump’s and Joe Biden’s incivility predicted a decrease in new Twitter followers. These results, therefore, support our theorizing that incivility depresses interest in hearing what a politician has to say.

## Study 2

The results of Studies 1a and 1b provided support in a real-world setting. However, by using real-world, field data, these studies sacrificed internal validity, leaving the possibility of alternative explanations. For example, poor media coverage or poor polling numbers might have led to both an increase in incivility and a decrease in followership. Furthermore, although Granger causality tests suggested incivility caused decreased interest, experimental studies are needed for direct causal inference. In addition, as we could not assess the political party affiliation of Twitter users in Studies 1a and 1b, we do not know if people reacted differently—possibly with greater interest—when a politician from the same party acts uncivilly. Study 2, therefore, experimentally manipulates the incivility of both Republican and Democratic politicians while also assessing participants’ party affiliation.^1^

### Method

#### Participants

Although our primary focus was incivility’s effect on levels of interest, to ensure we had enough power to detect a 3-way interaction in a 2(incivility vs. civility condition) × 2(Republican vs. Democratic politician) × 2 (Republican vs. Democratic participant) design, we calculated based on a pilot study (see OSM) that at least 800 participants were needed to find a small effect at 80% power. However, given the uneven distribution of Democratic and Republican participants (Levay et al., 2016) we would likely obtain, we targeted 1,500 participants from the Mechanical Turk website, recruiting only those previously vetted as high quality. The system collected data from 1,520 participants. However, 41 (3%) failed at least one of two attention checks (see OSM), leaving 1,479 participants (760 male, 717 female, and 2 did not indicate). The average age was 41.2 (*SD* = 12.8), with 76% identifying as white, 8% as Black, 6% as Hispanic/Latino, 8% as Asian, and 2% as Other. In addition, 37% were Republicans and 63% were Democrats. The average strength of party identification for Republicans was 4.51 out of 7 (*SD* = 1.71) and for Democrats was 4.61 (*SD* = 1.83).

#### Procedure

After a short demographics measure that also assessed political party affiliation, participants learned that in Part 1 of the study they would read statements made by several politicians and indicate whether they were interested in hearing more from each. Based on their answers, in Part 2, they would listen to a speech made by one of the politicians they indicated interest in hearing more from. Participants were then randomly assigned to read 5 tweets from either Republican or Democratic politicians (one tweet per politician) in a 2 (incivility vs. civility condition) × 2 (Republican vs. Democratic politician) × 2 (Republican vs. Democratic participant) between-subjects design. The variable of primary interest was the effect of the incivility manipulation on interest as it allowed us to test our hypothesis. We included the two additional variables to assess the generalizability of the effect, in particular, whether a party affiliation match (or mismatch) between the target politicians and participants might moderate the effects of the incivility manipulation. After reading each politician’s tweet, participants completed a manipulation check and indicated whether they were interested in hearing more from that politician. Then, participants learned there would not be a second part of the study and were debriefed.

#### Incivility Manipulation

Participants in the uncivil conditions read five uncivil tweets different politicians (either Democrat or Republican) had recently made (one tweet per politician). Participants in the civil conditions read a civil version of these same tweets, made by removing uncivil language while holding the message content constant (see OSM). Participants in the Republican Target condition read tweets from the following Republican politicians: Matt Gaetz, Majorie Taylor Greene, Lauren Boebert, Josh Hawley, and Jim Jordan. Participants in the Democrat Target condition read tweets from the following Democratic politicians: Jamie Raskin, Alexandria Ocasio-Cortez, Cori Bush, Beto O’Rourke, and Ilhan Omar.

#### Manipulation Check

Participants rated each tweet on civility on a scale from 0 (*not at all*) to 100 (*extremely*). To test whether any effects of the incivility manipulation were due to the tweet’s immaturity or lack of sophistication, participants also rated how “immature” and “unsophisticated” each tweet was. We averaged participants’ responses across the five targets for each variable (αs >.80).

#### Interest

Participants were asked

After reading this tweet by [politician’s name], in Part 2 of the study, would you prefer to hear more of what [politician’s name] has to say, or would you prefer to hear what a different [same political party] has to say?

Then participants indicated their preference to hear more from the target politician or a different politician from the same party as the target politician. We summed the number of times participants selected the target politician (0–5), which served as our outcome measure.

### Results

#### Manipulation Check

Participants judged the civil tweets (*M* = 50.42, *SD* = 25.62) to be more civil than the uncivil tweets (*M* = 33.54, *SD* = 25.13), *t*(1,477) = 12.79, *p* < .001, *d* = .67.

#### Effect of Incivility on Interest

To test the effect of incivility on follower interest, while also examining the potential moderating influence of co-partisanship, we conducted a 2 (incivility vs. civility condition) × 2 (Republican vs. Democratic politician) × 2 (Republican vs. Democratic participant) between-subjects analysis of variance (ANOVA), predicting interest in hearing more from the target politicians.^2^ The analysis yielded the predicted main effect of incivility, *F*(1, 1471) = 10.02, *p* = .002, albeit a small effect (*d* = .17); participants in the incivility conditions selected to hear from the target politicians significantly less, *M* = 1.59, *SD* = 1.50, than participants in the civility conditions, *M* = 1.89, *SD* = 1.54. The three-way interaction was nonsignificant, *F*(1, 1471) = 2.93, *p* = .207, *d* = .06, indicating that the effect of incivility was not different depending on whether the participant’s and the politicians’ party matched (see OSM for full factorial results table and figure).

#### Role of Incivility Versus Immaturity or Lack of Sophistication

To test whether the incivility of the tweets drove our effects as opposed to perceived immaturity or lack of sophistication, we conducted a mediation analysis (PROCESS Model-4) with incivility manipulation (0 = *civil*, 1 = *uncivil*) predicting interest mediated by civility ratings, and immaturity and unsophisticated ratings as covariates. The analysis yielded a significant indirect effect of civility ratings, *effect* = −.17, *SE* = .03, 95%CI [−.23, −.12] (see OSM for full results), indicating that participants’ judgments of how uncivil the targets were mediated the effect of the incivility manipulation on interest in hearing more of what the politicians had to say above and beyond any influence judgments of immaturity and lack of sophistication had.

### Discussion

Study 2 provided experimental support that incivility leads to less interest in what politicians have to say. Our analyses also indicated that co-partisanship did not moderate the effect of incivility, suggesting a general interest-depressing effect of incivility.

## Study 3

In Study 3, we tested whether incivility in speeches depresses interest in hearing what a politician has to say. Study 3 also tested our proposed model whereby incivility grabs attention but also evokes moral disapproval, with the negative influence of moral disapproval on interest suppressing any positive influence the attention-grabbing aspect of incivility might have on interest. After presenting participants with uncivil (vs. civil) speeches, we measured how much they found the speech to be attention-grabbing and how much they morally disapproved of its content and tested the mediating role each variable played in predicting interest in hearing more of what the politician had to say.

### Method

#### Participants

Like Study 2, we calculated our sample size based on the results of a pilot study (see OSM), determining that to have a 90% chance of detecting the main effect would require 577 participants.^3^ We recruited 604 Americans from Mechanical Turk (51% male, 49% female, and <1% non-binary) with a mean age of 40.21 (*SD* = 13). In addition, 34% were Republicans and 66% were Democrats.

#### Procedure

The study had a 2 (incivility vs. civility condition) × 2 (Republican vs. Democratic politician) × 2 (Republican vs. Democratic participant) between-subjects design. Participants read either a civil or a uncivil speech made by a fictitious Democratic or Republican politician. They then indicated how interested they were in reading more from that politician. Following this, they indicated how much they perceived the speech to be attention-grabbing and their level of moral approval or disapproval of the speech. A manipulation check item followed, along with a self-affirmation task to mitigate any negative affect, the manipulation might have caused. Then, they provided demographic information, including information about their political party. Finally, participants were debriefed and learned the research team had created the speech and the speaker.

#### Incivility Manipulation

Participants read the transcript of either a civilly or a uncivilly worded speech ostensibly by a Democratic or Republican politician adapted from Frimer and Skitka (2018). The speeches referenced the then-leaders of the Democratic and Republican caucuses in Congress: Chuck Schumer, Nancy Pelosi, Mitch McConnell, and Kevin McCarthy (see OSM).

#### Interest

Participants were asked, “How interested are you in reading about Senator Williams’ plans for infrastructure?” Responses were on 101-point scales anchored at 0 (*not at all*), 33 (*slightly*), 67 (*somewhat*), and 100 (*extremely*).

#### Mediators

*Moral disapproval* was measured using four questions (α = .94) assessing agreement with “I morally disapprove of the speech,”“The speech is offensive,”“The content of the speech is inappropriate,” and “I felt anger while reading the speech.”*Attention-grabbing* was measured with four questions (α = .88) assessing agreement with “The speech grabbed my attention,”“Its contents were intriguing,”“Its contents were memorable,” and “It has entertainment value.” Responses were on 101-point scales anchored at 0 (*not at all*), 33 (*slightly*), 67 (*somewhat*), and 100 (*extremely*).

#### Manipulation Check

Participants answered “How civil was Senator Williams’ speech?” on a 101-point scale anchored at 0 (*not at all*), 33 (*slightly*), 67 (*somewhat*), and 100 (*extremely*).

### Results

#### Manipulation Check

Participants judged the civil speech to be more civil, *M*=77.79, *SD*=22.26, than the uncivil speech, *M*=26.99, *SD*=24.76, *t*(599)=26.47, *p*<.001, *d*=2.16.

#### Effect of Incivility on Interest

**Figure 4:**
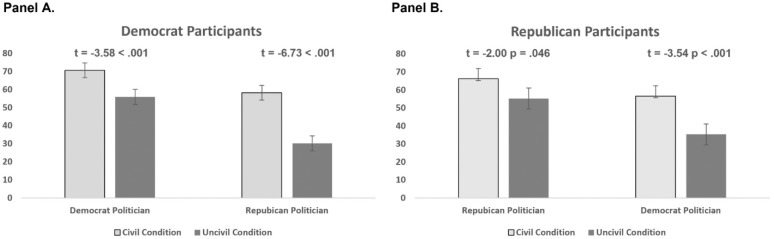
Graphs Depicting the 2 (Incivility vs. Civility Condition) × 2 (Republican vs. Democratic Politician) × 2 (Republican vs. Democratic Participant) Interaction, With Simple Effect Analyses Reported (Study 3) *Note.* Panel A represents Democratic participants’ interest levels depending on incivility condition and target politician. Panel B represents Republican participants’ interest levels depending on incivility condition and target politician.

A 2 (incivility vs. civility condition) × 2 (Republican vs. Democratic politician) x 2(Republican vs. Democratic participant) between-subjects ANOVA, predicting interest yielded the predicted main effect of incivility, *F*(1, 596) = 55.99, *p* < .001, *d* = .61. Participants in the incivility condition showed significantly less interest, *M* = 44.98, *SD* = 33.61, than participants in the civility conditions, *M* = 63.37, *SD* = 26.87. However, the three-way interaction was also significant, *F*(1, 596) = 5.62, *p* = .018, *d* = .19. Figure 4 presents the interaction with simple effects. As shown, there was some evidence that Republican participants were more lenient toward uncivil Republican politicians than Democratic participants were toward uncivil Democratic politicians. However, regardless of party pairings between participant and target, incivility depressed interest. Thus, these results indicate incivility depressed interest overall.

#### Mediation

**Figure 5:**
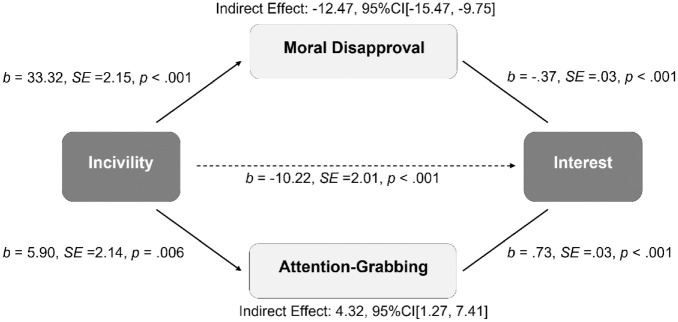
Mediation Model Finding That Incivility Depressed Follower Interest Because Participants Morally Disapproved of Incivility and This Effect Suppressed the Attention-Grabbing Nature of Incivility (Study 3)

We predicted the attention-grabbing nature of incivility would be overpowered by the aversive effects of people morally disapproving of the uncivil speech. A mediation analysis (PROCESS Model 4) with incivility (0 = *civil*, 1 = *uncivil*) predicting interest simultaneously mediated by *attention-grabbing* and *moral disapproval* found both mediators were significant. However, as predicted, the interest-piquing effect of *attention-grabbing* was suppressed by the interest-depressing effect of *moral disapproval*, yielding a negative overall effect of incivility on interest (Figure 5; see OSM for moderated mediation analyses).^4^

### Discussion

Study 3 replicated the interest-depressing effect of incivility on follower interest, this time in political speeches. The effect generalized across the political spectrum and was mediated by the moral disapproval that incivility drew, which suppressed its attention-grabbing nature.

## General Discussion

Four studies—two field and two experimental—found consistent evidence that incivility diminishes interest in hearing more from a politician. Our work both extends and challenges the existing literature on how people respond to incivility. A prevailing view has been that incivility is an effective tool for politicians because it grabs attention, which in turn garners interest in what a politician has to say. Our findings, however, provide a more nuanced perspective: Incivility is both attention-grabbing and morally distasteful, and in line with work showing the primacy of morality, the negative influence moral disapproval has on interest outweighs any positive influence gained by being attention-grabbing.

Of note, we found little evidence of moderation by co-partisanship, with incivility suppressing interest across the political spectrum. There was certainly evidence of in-party bias in our studies with Republicans showing more overall interest in hearing from Republican targets and Democrats showing more overall interest in hearing from Democratic targets, the effect of co-partisanship was *d = .*95 and *d* = .55 (Studies 2 and 3, respectively), while the effect of incivility was *d* = .17 and *d* = .61 (Studies 2 and 3, respectively). In other words, our results indicate individuals prefer to hear more from co-partisans, but when targets turn uncivil, interest dips regardless of which party that person is from. Such findings build on past work showing distasteful behaviors, such as incivility and immorality, often outweigh ingroup political biases (e.g., Feinberg et al., 2020; Frimer & Skitka, 2018).

### Potential Moderators

Although our results were consistent, a number of moderators might influence how incivility affects interest. Under certain circumstances, co-partisanship could moderate the effect of incivility on interest, even reverse it. For instance, those feeling strong animosity toward the opposing political party might embrace co-partisan’s incivility and wish to hear more. For them, incivility might elicit moral approval rather than disapproval, removing this barrier to interest. Similarly, those low on general agreeableness or moral identity, might not show decreased interest because incivility is less off-putting to them. Our samples might not have had enough of these individuals and therefore their influence was not apparent.

In addition, incivility in campaign ads might be less harmful to interest than incivility in other contexts, since people have come to expect such ads to feature uncivil attacks, and therefore may be more forgiving of this type of incivility. Likewise, incivility might impair interest in certain politicians less than others as people may come to expect incivility from certain politicians and become more inoculated against it. Indeed, a comparison of the number of potential followers lost by Trump and Biden in Studies 1a and 1b indicates that the effect of incivility was weaker for Trump than Biden.

Finally, it is unclear whether the effects of incivility on interest generalize beyond politics. Politics is likely a conservative test of our theorizing. Given the competitive nature of politics, people might be more forgiving of incivility. In less ruthless contexts, people might be less forgiving and feel even less interest in hearing from an uncivil person (see OSM for evidence that incivility diminishes interest in the domain of online media).

### Limitations and Future Directions

Although our studies provided converging evidence, there are a number of limitations and future directions. Our work was largely American-centric, mostly examining responses to American politicians by American participants. Whether incivility depresses interest in other countries is unknown and needs exploration. In addition, our measures of interest across studies did not require strong behavioral commitment. In Study 1, participants followed a politician on Twitter, in Study 2 participants’ indications of interest ostensibly resulted in them listening to a short speech by a target, and in Study 3 participants only reported their interest. It is unclear whether incivility’s effects on interest might differ when expressing interest requires more behavioral commitment, for instance attending a speech or signing up for a politician’s newsletter—something future research might explore. Relatedly, in our experiments, we measured perceptions of civility as a manipulation check, including asking about it prior to assessing interest in Study 2. Although in Study 1 we did not measure civility and in Study 3 we measured it after interest had been reported, it is still important to note that measuring civility prior to interest could have influenced responses, possibly due to concerns with impression management—something strong behavioral measures could also help address.

Our results suggest politicians should act more civilly if they wish to be influential, yet there might be other functions of incivility that offset the loss of interest. Politicians might willingly impair interest if their uncivil rhetoric poses even more damage to political opponents’ reputations. In addition, if incivility depresses interest in politics in general, incumbents worried the electorate is turning on them might benefit by using incivility to minimize voter turnout.

Relatedly, it is unclear whether incivility minimizes interest in the politician acting uncivilly, or in that politician’s party in general. Research on the radical flank effect (Simpson et al., 2022), wherein radical groups in social movement lead observers to support more moderate groups of that same movement and suggests uncivil politicians could make civil politicians in their party look better via contrast effects. Thus, even if uncivil politicians decrease the public’s interest in them, they may be building interest in their party members. Future research should explore these important questions relating to how incivility influences interest.

## Supplemental Material

sj-docx-1-spp-10.1177_19485506221136182 – Supplemental material for Incivility Diminishes Interest in What Politicians Have to Say Click here for additional data file. Supplemental material, sj-docx-1-spp-10.1177_19485506221136182 for Incivility Diminishes Interest in What Politicians Have to Say by Matthew Feinberg and Jeremy A. Frimer in Social Psychological and Personality Science

## Notes

1. See OSM for an additional experimental study showing incivility’s interest-depressing effects.

2. For Studies 2 and 3, we also created a “party match” variable dividing participants into those who matched and those who mismatched the political party of the target politicians. See OSM for results of incivility condition by party match analyses.

3. Since Study 2’s results found co-partisanship was nonsignificant, our sample selection focused on the main effects of incivility.

4. It is possible the strong effects for *moral disapproval* were due to certain items in that composite being psychologically close to incivility. We examine this possibility in the OSM.
