# Supplementary material for: Incivility Diminishes Interest in What Politicians Have to Say
Source: Soc Psychol Personal Sci. 2022 Nov 16;14(7):787–95. doi: 10.1177/19485506221136182 (PMC10396794; doi:10.1177/19485506221136182)
Supplement: sj-docx-1-spp-10.1177_19485506221136182 – Supplemental material for Incivility Diminishes Interest in What Politicians Have to Say [file sj-docx-1-spp-10.1177_19485506221136182.docx]

**Online Supplemental Materials**

**Study 1a &b**

**Linear vs. Curvilinear relationship between incivility and followers**

It may be that the relationship between incivility and followership is curvilinear rather than linear. The regression analyses presented below (Supplementary Table 1) test this possibility using both the Trump and Biden data.

**Supplementary Table 1.** Test of linear versus curvilinear effects in Studies 1a (Trump) and 1b (Biden)

***Trump***

*Linear Model Predicting “Followers Tomorrow”*

|  |  |  |  |  | **Correlations** | | | |
| --- | --- | --- | --- | --- | --- | --- | --- | --- |
| **Predictor** | ***b*** | ***SE*** | ***t*** | **p-value** | **Zero-Order** | **Partial** | | **Part** |
| Constant | 49809.371 | 3777.829 | 13.185 | p<.001 |  | |  |  |
| Follower (today) | 1 | 0 | 23265.129 | p<.001 | 1 | | 1 | .996 |
| Incivility | -61962.332 | 16425.124 | -3.772 | p<.001 | .088 | | -.085 | 0 |

*Curvilinear Model Predicting “Followers Tomorrow”*

|  |  |  |  |  | **Correlations** | | | |
| --- | --- | --- | --- | --- | --- | --- | --- | --- |
| **Predictor** | ***b*** | ***SE*** | ***t*** | **p-value** | **Zero-Order** | **Partial** | | **Part** |
| Constant | 42457.985 | 2692.933 | 15.766 | p<.001 |  | |  |  |
| Follower (today) | 1 | 0 | 23304.847 | p<.001 | 1 | | 1 | .999 |
| Incivility.Squared | -100073.13 | 36363.65 |  | p=.006 | .033 | | -.062 | 0 |

***Biden***

*Linear Model Predicting “Followers Tomorrow”*

|  |  |  |  |  | **Correlations** | | | |
| --- | --- | --- | --- | --- | --- | --- | --- | --- |
| **Predictor** | ***b*** | ***SE*** | ***t*** | **p-value** | **Zero-Order** | **Partial** | | **Part** |
| Constant | 101003.662 | 21233.939 | 4.757 | p<.001 |  | |  |  |
| Follower (today) | 1 | .001 | 1125.981 | p<.001 | 1 | | 1 | .951 |
| Incivility | - 289202.875 | 111388.089 | -2.596 | p=.01 | .031 | | -.13 | -.002 |

*Curvilinear Model Predicting “Followers Tomorrow”*

|  |  |  |  |  | **Correlations** | | | |
| --- | --- | --- | --- | --- | --- | --- | --- | --- |
| **Predictor** | ***b*** | ***SE*** | ***t*** | **p-value** | **Zero-Order** | **Partial** | | **Part** |
| Constant | 73543.185 | 17591.535 | 4.181 | p<.001 |  | |  |  |
| Follower (today) | 1 | .0001 | 1118.884 | p<.001 | 1 | | 1 | .948 |
| Incivility.Squared | -496600.973 | 240159.692 | -2.068 | p=.039 | .0319 | | -.104 | -.002 |

As shown, the regression results are stronger for the linear model, suggesting this model fit the data better than a curvilinear model.

**Threshold where incivility begins to decrease followers**

It is unclear at what point incivility starts to impact followership. To explore that we conducted regression analyses examining the influence of incivility on new followers for varying levels of incivility (ranging from 0 to .7). Below we present the 95% confidence intervals for new followers added at each level of incivility. Note: Baseline is the number of new followers expected when incivility is at 0.

**Supplementary Table 2.** Data showing the relationship between incivility ratings and new followers added. (Study 1a)

***Trump***

|  | **New Followers** | | **Baseline** |  |
| --- | --- | --- | --- | --- |
| **Incivility** | **Lower** | **Upper** | **Lower** | **Upper** |
| 0 | 47478 | 60884 | 47478 | 60884 |
| 0.1 | 44544 | 52211 | 47478 | 60884 |
| 0.2 | 40312 | 44835 | 47478 | 60884 |
| 0.3 | 32746 | 40793 | 47478 | 60884 |
| 0.4 | 24044 | 37888 | 47478 | 60884 |
| 0.5 | 15148 | 35178 | 47478 | 60884 |
| 0.6 | 6193 | 32525 | 47478 | 60884 |
| 0.7 | -2786 | 29897 | 47478 | 60884 |

**Supplementary Materials Figure 1.** Graphical depiction of the relationship between incivility ratings and new followers added. (Study 1a)

**Supplementary Table 3.** Data showing the relationship between incivility ratings and new followers added. (Study 1b)

***Biden***

|  | **New Followers** |  | **Baseline** |  |
| --- | --- | --- | --- | --- |
| **Incivility** | **Lower** | **Upper** | **Lower** | **Upper** |
| 0 | 62628 | 124752 | 62628 | 124752 |
| 0.1 | 55837 | 89860 | 62628 | 124752 |
| 0.2 | 35755 | 68258 | 62628 | 124752 |
| 0.3 | 1349 | 60981 | 62628 | 124752 |
| 0.4 | -36197 | 56843 | 62628 | 124752 |
| 0.5 | -74467 | 53429 | 62628 | 124752 |
| 0.6 | -112999 | 50277 | 62628 | 124752 |
| 0.7 | -151653 | 47248 | 62628 | 124752 |

**Supplementary Materials Figure 2.** Graphical depiction of the relationship between incivility ratings and new followers added. (Study 1b)

As shown above, for Trump, the number of new followers added drops below baseline at an incivility level of about .2. For Biden, this number is about .3. This means that for Trump, when his incivility reached a level of .2, he began to lose followers, and for Biden when his incivility reached a level of .3, he began to lose followers. Of note: any difference between Trump and Biden should be interpreted cautiously given the differences in sample sizes between the two.

**Study 2**

**Stimuli**

**Uncivil Republican Tweets**


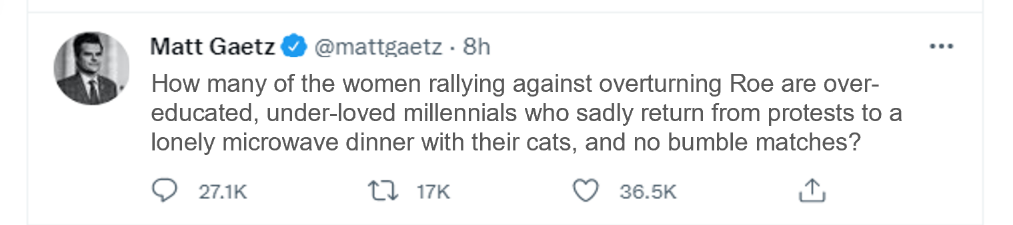


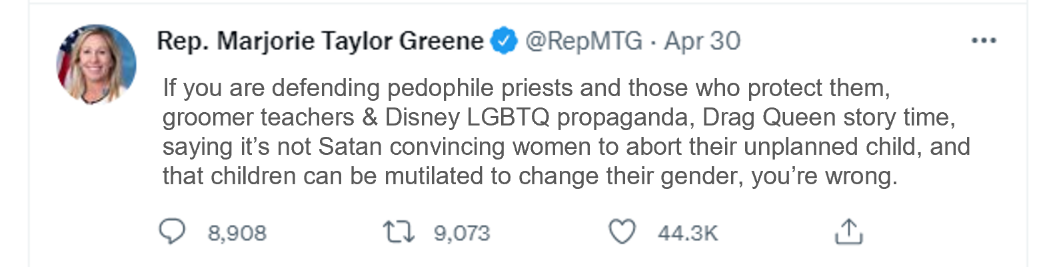


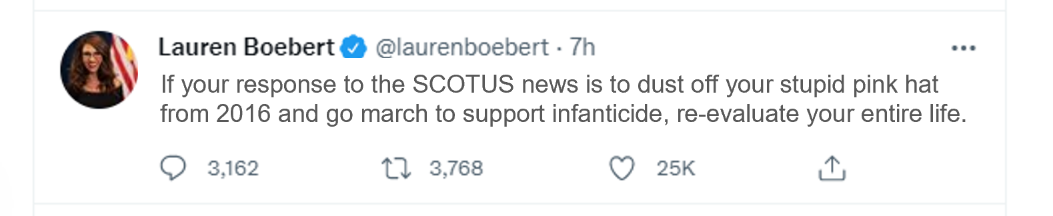


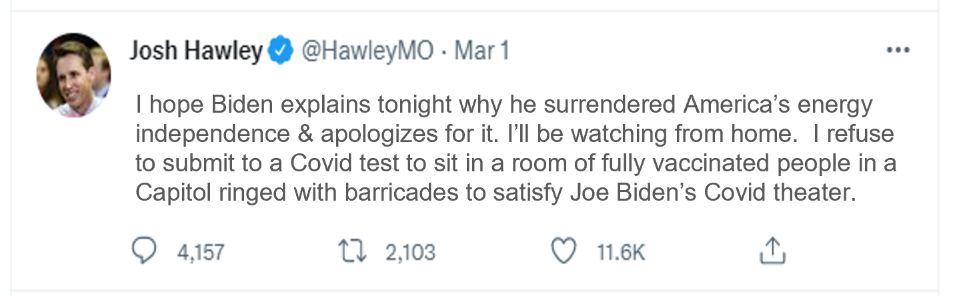


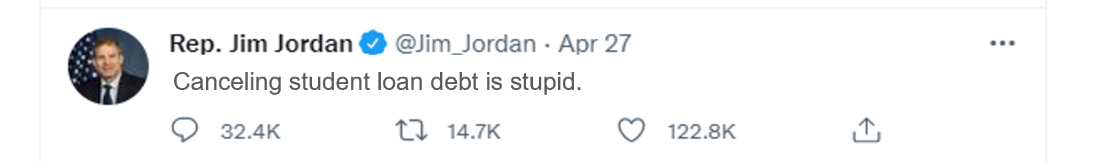


**Civil Republican Tweets**


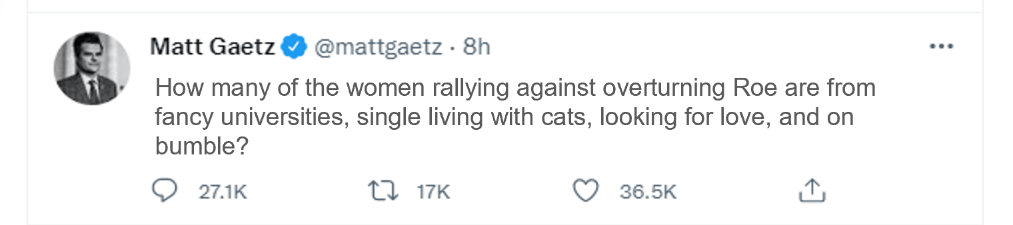


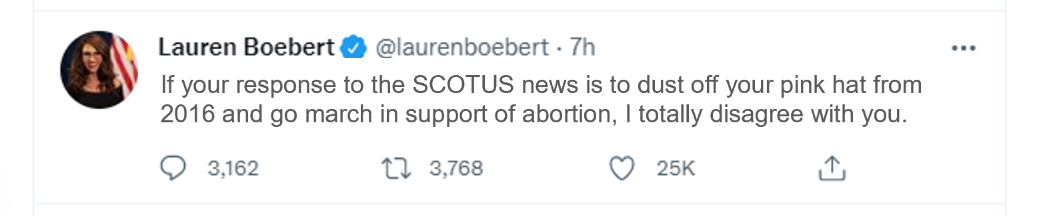


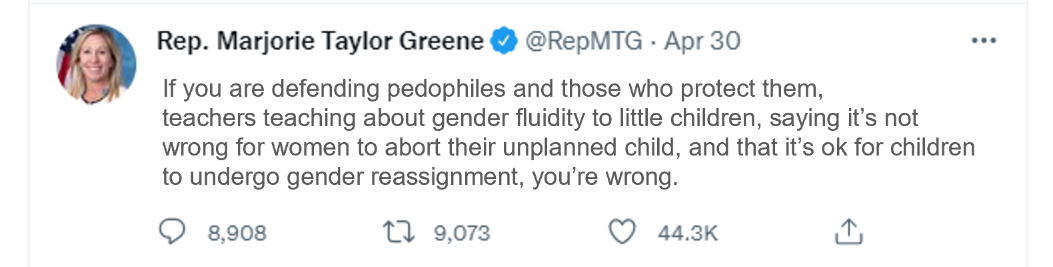


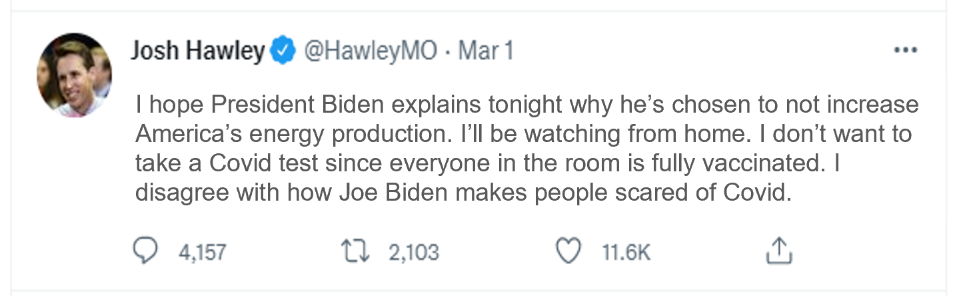


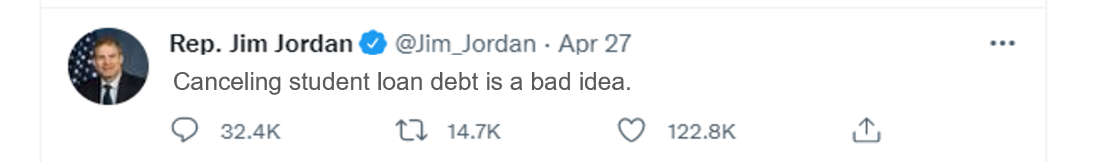


**Uncivil Democrat Tweets**


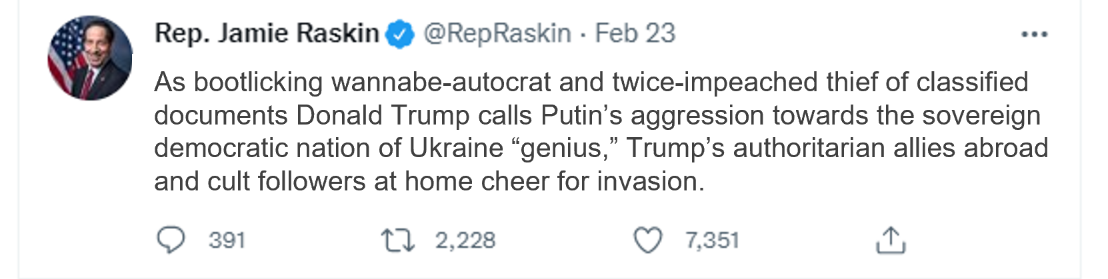


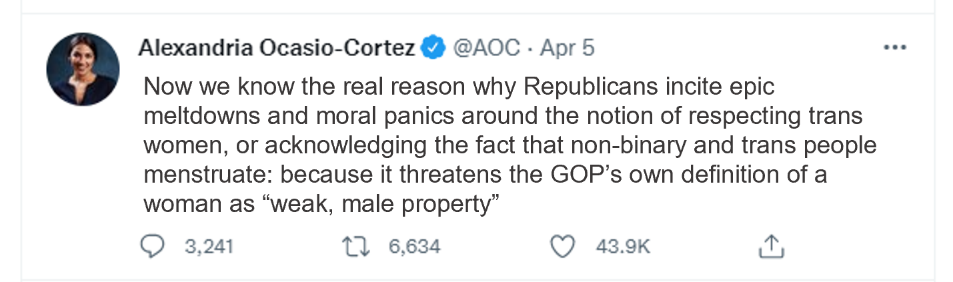


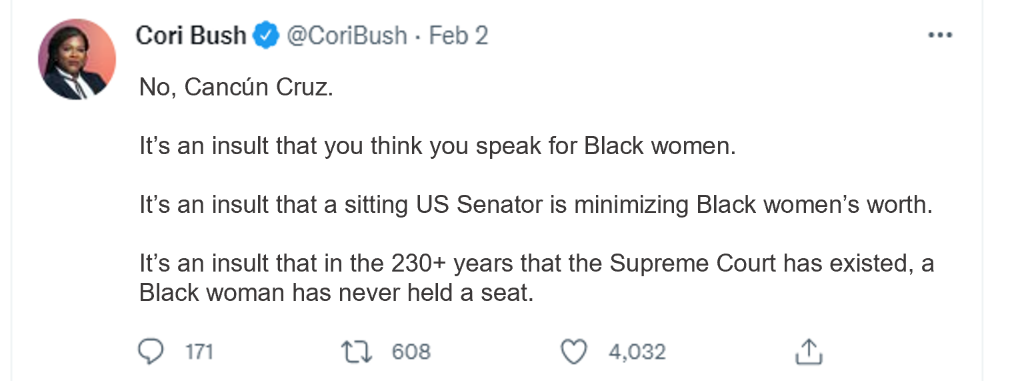


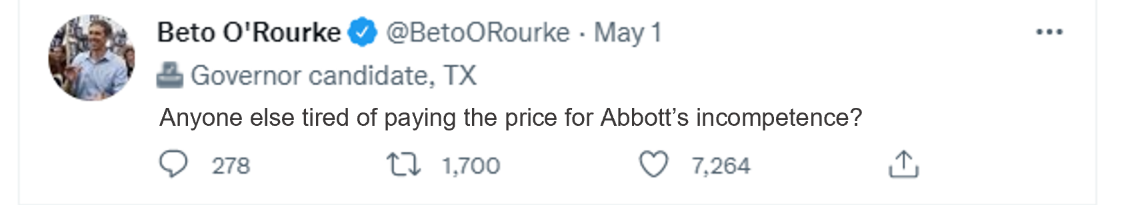


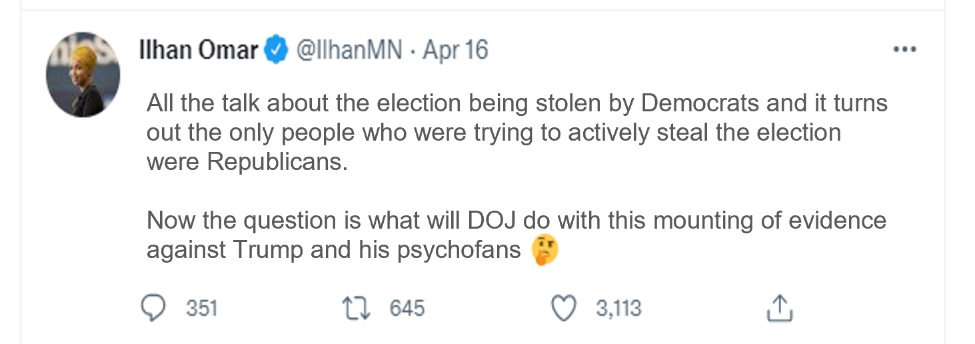


**Civil Democrat Tweets**


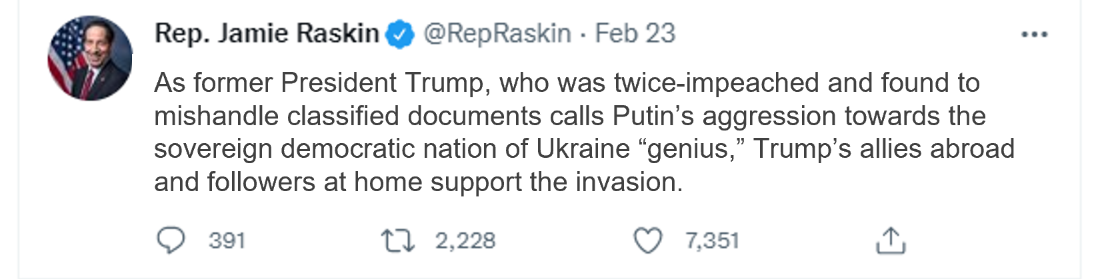


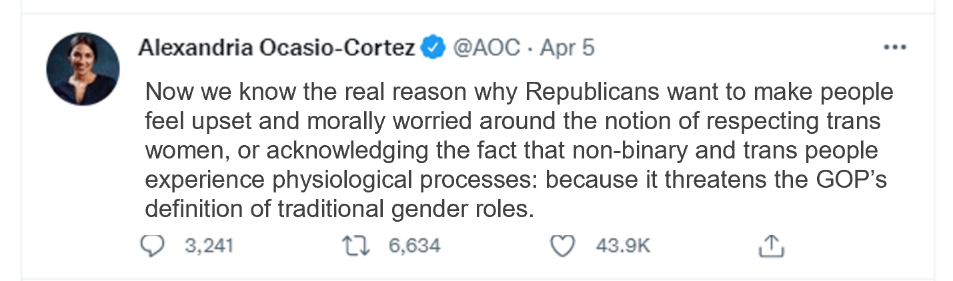


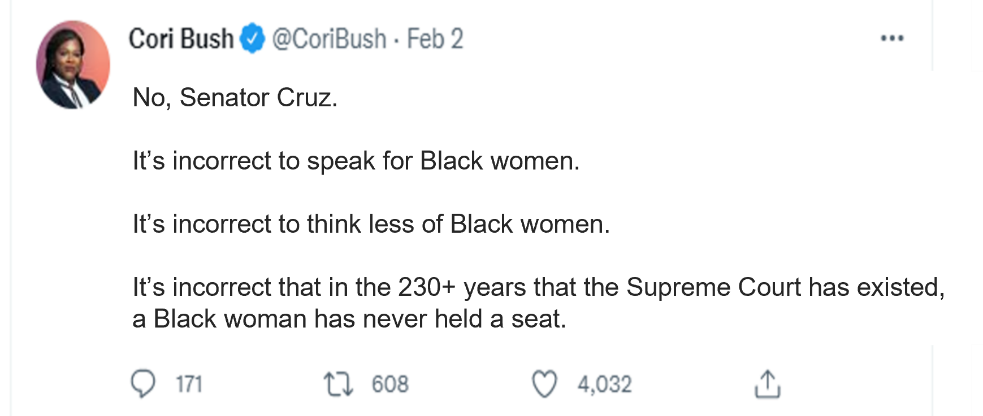


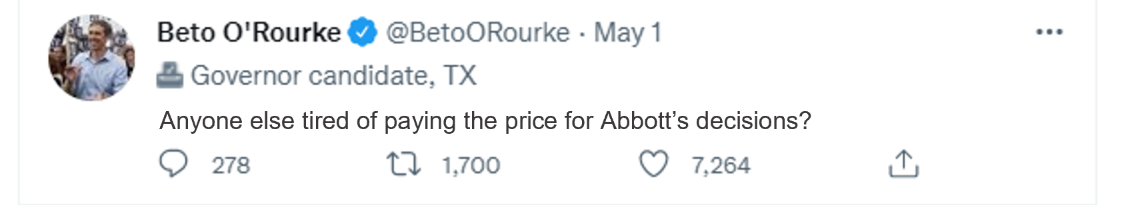


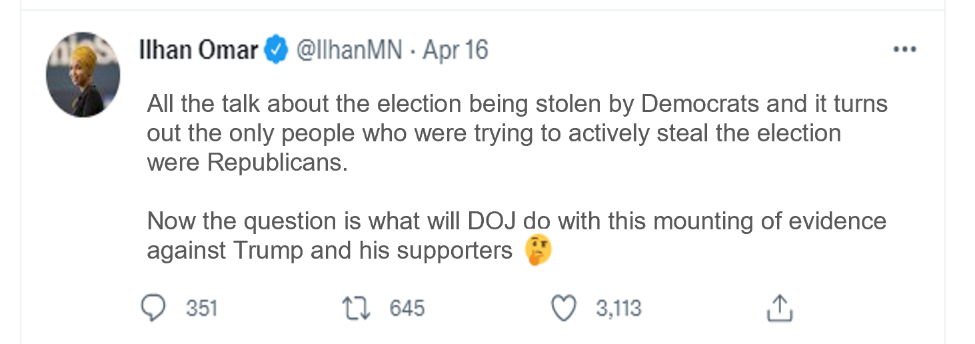


**Attention Check Questions**

The following were the items we used as attention check measures.

1. To ensure you are attending to each question carefully, answer three for this question.

Response options were 1 to 7.

1. Based on your experience with the study so far, what would you say it has been about?

Response options were:

Rating the quality of companies

Rating different cultures

Choosing among political tweets

Choosing among types of animals

Describing how people fall in love

Describing how people choose their friends

Selecting your favorite food

Selecting your favorite pets

Identifying different fonts

Identifying different colors

For question 1, participants failed the attention check if they provided any response besides “3”.

For question 2, participants failed the attention check if they provided any response besides “Choosing among political tweets”.

**Full Factorial ANOVA results**

Below we present the results of the full-factorial ANOVA entering the incivility manipulation, participant’s political party, and target politician’s political party as predictors of participants’ interest in hearing more.

**Supplementary Table 4.** Full Factorial ANOVA results (Study 2)

| **Predictor** | **F value** | **P value** | **Effect Size** |
| --- | --- | --- | --- |
| Incivility Manipulation | *F*(1, 1471) = 10.02 | *p* = .002 | *d* = .17 |
| Participant’s Party | *F*(1, 1471) = .85 | *p* = .357 | *d* = .06 |
| Politician’s Party | *F*(1, 1471) = 3.15 | *p* = .076 | *d* = .09 |
| Incivility x Participant | *F*(1, 1471) = 1.94 | *p* = .164 | *d* = .06 |
| Incivility x Politician | *F*(1, 1471) = 5.96 | *p* = .015 | *d* = .13 |
| Participant x Politician | *F*(1, 1471) = 328.65 | *p* < .001 | *d* = .95 |
| Incivility x Participant x Politician | *F*(1, 1471) = 1.56 | *p* = .207 | *d* = .06 |

As described in the main text, these results suggest there was no moderation by co-partisanship. Rather, there was only an effect of incivility on participants’ levels of interest – however see next section.

**Examination of the (null) 3-way ANOVA interaction between participants’ political party, targets’ political party, and incivility manipulation.**

Although the 3-way ANOVA interaction was not significant (*F*(1,1471)=2.93, *p*=.207, *d*=.06), how our incivility manipulation influenced both Democrat and Republican participants as they indicated interest in hearing more from Democrat and Republican politicians may be of interest to readers. For this reason, below in Supplementary Figure 5, we separately depict participants’ (Democrats’ and Republicans’, separately) interest as a function of the politicians’ incivility and political party. As shown, for Democrats, there was consistently less support for target politicians – either Democrats or Republican targets – who were uncivil. Interestingly, for Republicans, there was less support for Republican politicians who were uncivil, but there was no significant difference for Democrat politicians, suggesting Republican participants were not influenced by the incivility of Democrat targets. However, since the omnibus 3-way interaction was not significant, and we do not find parallel results in any of our other studies, we believe this this singular null finding should be interpreted with caution.

**Supplementary Figure 5.** Graphical depiction of the full factorial ANOVA examining the interaction between participants’ political party, targets’ political party, and incivility manipulation, separated by Democrat (Panel A) and Republican (Panel B) participants.


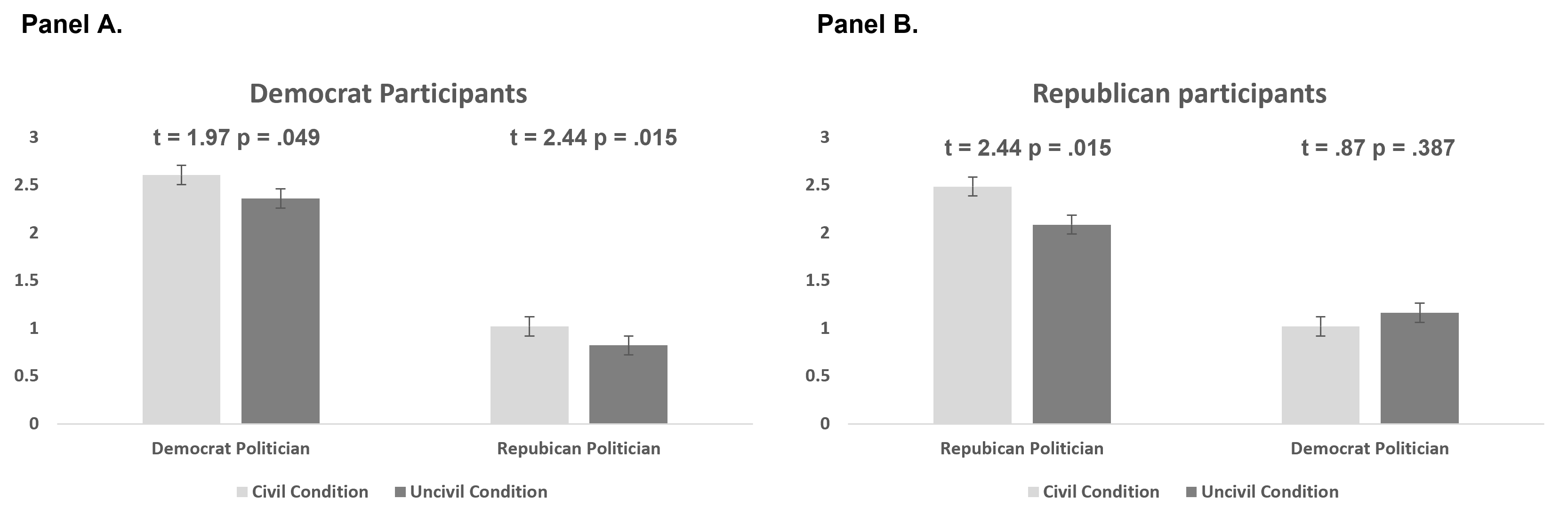


**2-way interactions looking at “party match vs. mismatch” x Incivility Manipulation**

An alternative means for examining the moderating role of co-partisanship could be to create a variable for each participant indicating whether the targets they looked at were of the same party or of the opposing party. Below we report the results of an ANOVA where we entered incivility manipulation and whether there was a party match or mismatch (between the participant and the target politician) as predictors of participant interest in hearing more from the politician. These analyses involved creating a new variable that indicates whether the participant and the target politician were from the same party (coded as 1) or from different parties (coded as 0).

**Supplementary Table 5.** 2-way interactions looking at “party match vs. mismatch” x Incivility Manipulation (Study 2)

| **Predictor** | **F value** | **P value** | **Effect Size** |
| --- | --- | --- | --- |
| Incivility Manipulation | *F*(1, 1475) = 12.68 | *p* < .001 | *d* = .19 |
| Political Party Match | *F*(1, 1475) = 369.72 | *p* < .001 | *d* = 1.00 |
| Incivility x Party Match | *F*(1, 1475) = .38 | *p* = .537 | *d* = .03 |

The results of these analyses coincide with the findings of the full model using all three factors – i.e., there was not moderation by co-partisanship.

**Mediation Results**

To isolate the mediating role of civility ratings from the influence of immaturity and sophistication ratings, we conducted a mediation analysis (Process Model 4) entering civility as the mediator and immaturity and sophistication ratings as covariates. The results from this analysis are presented in Supplementary Figure 3 below. As shown, even when controlling for immaturity and lack of sophistication, civility ratings were a significant mediator of the relationship between incivility and interest (i.e., targets chosen).

**Supplementary Figure 3.** Indirect effect of participants’ civility ratings, controlling for immaturity and lack of sophistication. (Study 2)

*
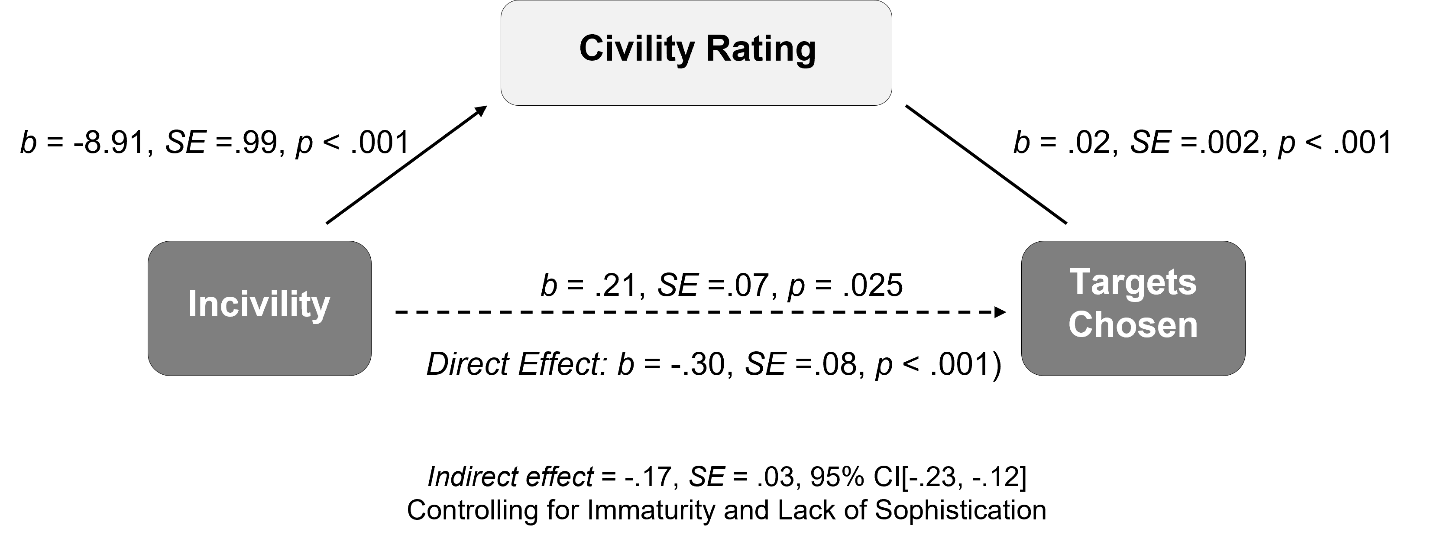
*

We also explored the unique influence of civility ratings by conducting a simultaneous mediation analysis (Process Model 4) entering civility, immaturity, and lack of sophistication as mediators. The results of this analysis are presented below in Supplementary Figure 4. As shown, the results for this analysis parallel the above – i.e., civility ratings were a unique mediator for the relationship between incivility and interest. Additionally, an examination of the mediating role of immaturity and lack of sophistication found that the indirect effect of immaturity was not significant (indirect effect: -.07, 95%CI[-.16, .02]), but lack of sophistication was significant (indirect effect: -.11, 95%CI[-.20, -.04]). This latter finding suggests that, along with civility perceptions, perceptions that a politician’s Tweets lacked sophistication also (independently) helped explain the effects we found.

Additionally, a closer look at the c’ value (*b* = .21, *SE* = .07, *p* = .025) of the mediation analyses reveals that the direction of the effect for the relationship between incivility and targets chosen flipped. This suggests that when one removes the influence of incivility ratings (and immaturity and lack of sophistication), then the remaining influence of the incivility manipulation actually results in increased interest. In other words, if a politician engages in uncivil behavior, but manages to have such behavior not be viewed as uncivil (or immature or unsophisticated), then that behavior can be a means for increasing interest in what the politician has to say.

**Supplementary Figure 4.** Simultaneous mediation analyses entering civility ratings, immaturity, and lack of sophistication as mediators. (Study 2)
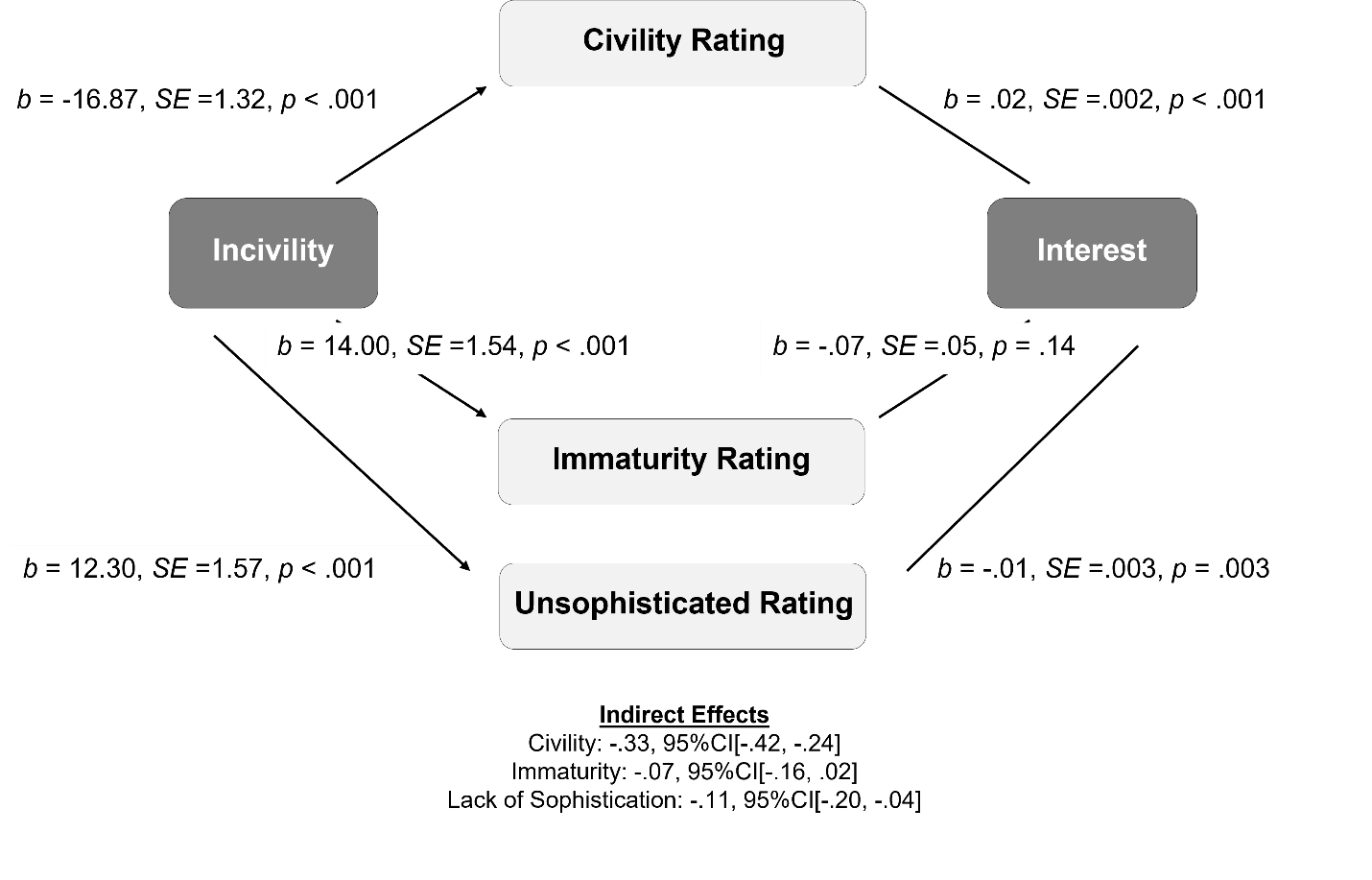


**Study** **3**

**Stimuli**

The instructions read, “Please read the following speech by a Republican [Democratic] member of Congress, Senator Williams.”

*Civil speech*.

We believe that it’s time to invest in our infrastructure—bridges, roads, airports. Our Democrat [Republican] colleagues have put forward a proposal to make this happen. Independent experts looked at the proposal and predicted that 3 million people would lose their jobs. And the experts say that the projects would be over-budget. Mr. Schumer, Ms. Pelosi [Mr. McConnell, Mr. McCarthy] and the rest of our friends and colleagues from across the aisle: we appreciate that we share an interest in improving our nation’s infrastructure. However, we respectfully disagree about how to make it happen. Our proposal would go further to create working class jobs and get America rebuilt under-budget. Let’s debate this in the open. We are interested in what you have to say.

*Uncivil speech*.

I know for certain that it’s time to invest in our infrastructure—bridges, roads, airports. Radical Democrats [Republicans] have put forward a proposal to make this happen. Independent experts looked at your proposal and predicted that 3 million people would lose their jobs. And the experts say that the projects would be over-budget. Shameful! Chuck, Nancy [Mitch, Kevin], and the rest of you slimy Democrats [Republicans] across the aisle: we appreciate that you at least pretend to share an interest in improving our nation’s infrastructure. However, you are dead wrong about how to make it happen. My proposal would go further to create working class jobs and get America rebuilt under-budget. Sure. We can debate this in the open. I’m willing to let you run your mouths.

**Full Factorial ANOVA Results**

The 3-way ANOVA interaction examining the effects of incivility manipulation, participant’s political party, and target politician’s political party on participants’ interest was significant. Figure 4 in the main text provides a graphical depiction of this 3-way interaction. However, below in Supplementary Table 5, we provide the full factorial results of this analysis. As described in the main text, unlike Study 2 there was a significant 3-way interaction.

**Supplementary Table 5.** Full factorial ANOVA results looking at the effects of incivility manipulation, participant’s political party, and target politician’s political party. (Study 3).

| **Predictor** | **F value** | **P value** | **Effect Size** |
| --- | --- | --- | --- |
| Incivility Manipulation | *F*(1, 596) = 55.99 | *p* < .001 | *d* = .61 |
| Participant’s Party | *F*(1, 596) = .03 | *p* = .874 | *d* = .01 |
| Politician’s Party | *F*(1, 596) = .72 | *p* = .396 | *d* = .06 |
| Incivility x Participant | *F*(1, 596) = 1.07 | *p* = .302 | *d* = .09 |
| Incivility x Politician | *F*(1, 596) =.09 | *p* = .767 | *d* = .02 |
| Participant x Politician | *F*(1, 596) = 45.52 | *p* < .001 | *d* = .55 |
| Incivility x Participant x Politician | *F*(1, 596) = 5.62 | *p* = .018 | *d* = .19 |

**Test of Moderated Mediation**

It may be that the mediation results we found where moral disapproval suppresses the influence of attention-grabbing only applies to participants of one political party and not the other. To explore this possibility, we separated participants into Democrats and Republicans and then ran moderated mediation analyses for each group, examining the mediating roles of moral disapproval and attention-grabbing in explaining effects of the incivility condition x target politician’s party on levels of interest (i.e., Incivility Condition x Politician’s Party 🡪 Moral Disapproval/Attention Grabbing 🡪 Interest). The results of these analyses are reported below in Supplementary Tables 6 & 7.

**Supplementary Table 6.** Test of moderated mediation examining the mediating influence of moral approval and attention-grabbing in explaining the effect of the incivility manipulation on interest for Democrat participants. (Study 3)

*Democratic Participants*

Incivility Condition 🡪 Moral Approval 🡪 Interest

Politician’s Party Effect BootSE BootLLCI BootULCI

Democratic -7.7666 1.5607 -11.0124 -4.9804

Republican -16.9148 2.6690 -22.3406 -11.9542

Index of moderated mediation:

Index BootSE BootLLCI BootULCI

Politician’s Party -9.1482 2.0648 -13.4416 -5.4387

Incivility Condition 🡪 Attention-Grabbing 🡪 Interest

Politician’s Party Effect BootSE BootLLCI BootULCI

Democratic 1.0550 2.4743 -3.8001 5.8955

Republican 3.7283 2.7447 -1.5042 9.2186

Index of moderated mediation (difference between conditional indirect effects):

Index BootSE BootLLCI BootULCI

Politician’s Party 2.6733 3.7062 -4.5550 10.0445

This result indicates that for the Democratic participants, moral disapproval mediated the effect of incivility in decreasing interest for both Democratic and Republican targets. However, the significant index of moderated mediation for moral disapproval suggests this mediation was stronger when the target politician was a Republican. The results also show that attention-grabbing was a non-significant mediator for both Democratic and Republican targets, and the non-significant index moderated mediation suggests that attention-grabbing’s influence was not different when the target was a Democrat or a Republican.

**Supplementary Table 7.** Test of moderated mediation examining the mediating influence of moral approval and attention-grabbing in explaining the effect of the incivility manipulation on interest for Republican participants. (Study 3)

*Republican Participants*

Incivility Condition 🡪 Moral Approval 🡪 Interest

Politician’s Party Effect BootSE BootLLCI BootULCI

Democratic -13.7117 2.7183 -19.3651 -8.7421

Republican -9.8400 2.5339 -15.3262 -5.3929

Index of moderated mediation:

Index BootSE BootLLCI BootULCI

Politician’s Party 3.8717 2.7379 -1.5566 9.2259

Incivility Condition 🡪 Attention-Grabbing 🡪 Interest

Politician’s Party Effect BootSE BootLLCI BootULCI

Democratic 4.9903 3.7196 -2.3687 12.4332

Republican 7.8499 3.7500 .3228 15.1807

Index of moderated mediation:

Index BootSE BootLLCI BootULCI

Politician’s Party 2.8596 5.2197 -7.0283 13.2706

This result indicates that for the Republican participants, moral disapproval mediated the effect of incivility in decreasing interest for both Democratic and Republican targets. The non-significant index of moderated mediation for moral disapproval suggests the mediating role of moral disapproval was not different when the target was a Democrat or a Republican. Additionally, the results show that attention-grabbing was a significant mediator only when the target was a Republican. However, the non-significant moderated mediation suggests that attention-grabbing’s influence was not different when the target was a Democrat or a Republican.

**2-way interactions looking at “party match vs. mismatch” x Incivility Manipulation**

We used the same procedure as in Study 2 described above to examine the possible influence of co-partisanship. Results of this analysis are reported below in Supplementary Table 8. As above, these analyses involved creating a new variable that indicates whether the participant and the target politician were from the same party (coded as 1) or from different parties (coded as 0).

**Supplementary Table 8.** Examination of the 2-way interactions looking at “party match vs. mismatch” x Incivility Manipulation. (Study 3)

| **Predictor** | **F value** | **P value** | **Effect Size** |
| --- | --- | --- | --- |
| Incivility Manipulation | *F*(1, 600) = 68.61 | *p* < .001 | *d* = .68 |
| Political Party Match | *F*(1, 600) = 54.99 | *p* < .001 | *d* = .61 |
| Incivility x Party Match | *F*(1, 600) = 6.93 | *p* = .009 | *d* = .21 |

In line with the full 3-factor ANOVA results, this alternative means for testing the influence of co-partisanship yielded a parallel result – in this study co-partisanship moderated the effect of the incivility manipulation.

**Mediation by most face-valid item from the moral disapproval composite**

Some items in the moral disapproval composite might be seen as psychologically close to incivility (e.g., “The speech is offensive”), assessing incivility rather than moral disapproval. If so, this raises the possibility that this psychological closeness (a) explained why the moral disapproval composite was such a strong mediator, and (b) explained why the moral disapproval composite suppressed the mediating influence of the attention-grabbing composite.

To examine this possibility, we conducted mediation analyses (Process Model 4) using the most face-valid item from the moral disapproval composite (“I morally disapprove of the speech”), which participants were unlikely to see as a measure of incivility rather than a measure of moral disapproval. First, we found that this item alone mediated the relationship between the experimental condition and participants’ interest, CI 95%[-13.74, -7.25]. This result suggest that feelings of moral disapproval mediated the relationship. Second, we found that when this item and the attention-grabbing composite were entered as simultaneous mediators, we still found the hypothesized effect (See Supplementary Figure 6 below).

**Supplementary Figure 6.** Indirect effect of incivility on interest via moral disapproval and attention-grabbing using only the most face-valid moral disapproval item. (Study 3)


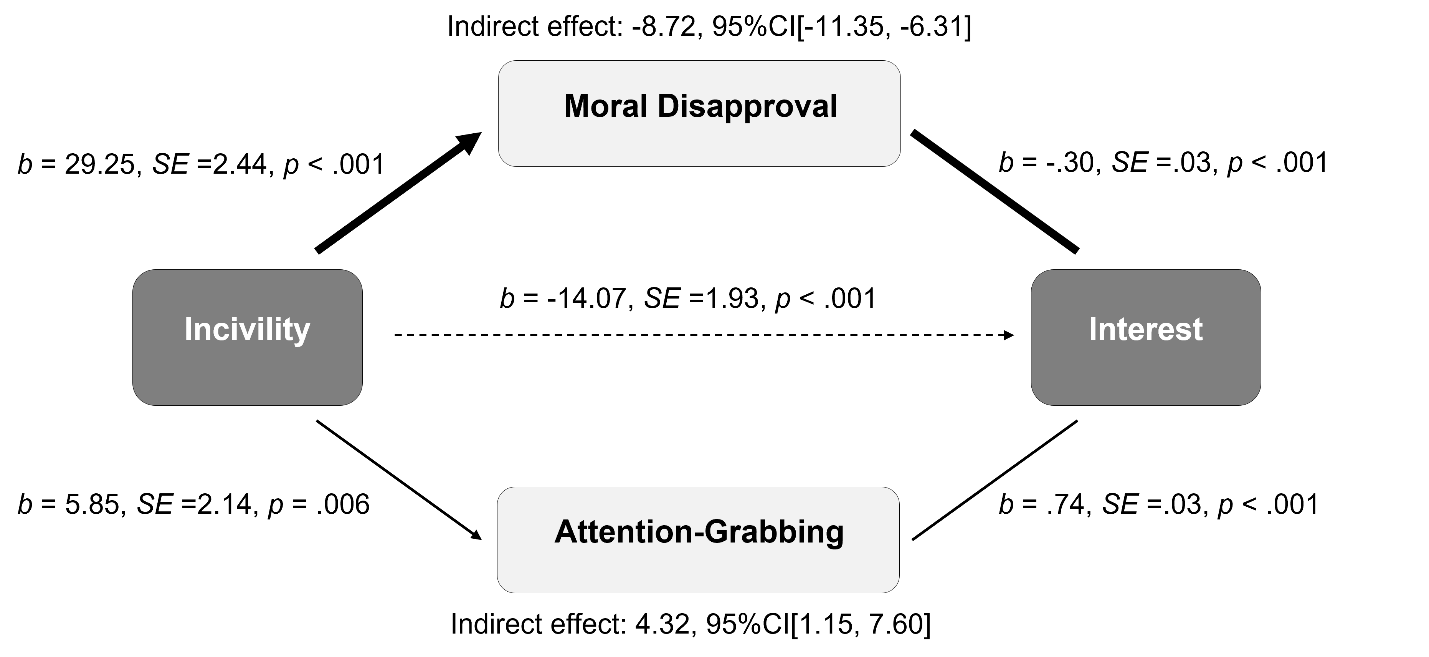


These results, using the most face-valid moral disapproval item, suggest that the larger mediation results we found, where moral disapproval suppressed the influence of attention-grabbing, were unlikely due to psychological overlap between the moral disapproval composite and notions of incivility.

**Supplemental Study 1 – Pilot Study**

**Method**

**Participants**. We recruited a sample large enough to detect a small effect (*r* ~ .1) with 80% power and a 2-tailed test. The sample was 530 participants from Amazon’s Mechanical Turk with 59% of participants being male, 41% being female and being <1% non-binary.^^[[1]](#footnote-1)^^ The average age was 37.28 years old (*SD* = 12.30). Additionally, 38% were Republicans and 62% were Democrats. The study was conducted in April 2019 when Donald Trump was President and Bernie Sanders was among the frontrunners competing for the Democratic nomination for the presidency in 2020.

**Procedure**. Participants were randomly assigned to read either a civil or uncivil pair of tweets by either President Donald Trump or Senator Bernie Sanders in a 2(incivility vs. civility condition) x 2(Republican vs. Democratic politician) x 2(Republican vs. Democratic participant) between-subjects design. After indicating how interested they were in reading more tweets from their assigned target politician, participants completed a manipulation check, a self-affirmation induction to reduce possible negative affect, and reported demographics, which included information about participants’ political orientation and Twitter habits. Lastly, we debriefed participants explaining the purpose of the study and explaining that the text attributed to the target politicians were made up.

***Incivility manipulation.*** Participants were randomly assigned to read a pair of civil (*n* = 281) or uncivil (*n* = 249) tweets ostensibly by President Trump (*n* = 267) or Senator Bernie Sanders (*n* = 263; see Stimuli below). The instructions in the Trump [Sanders] condition read, “Please review the following tweet wherein President Donald Trump [Senator Bernie Sanders] addresses Democratic [Republican] leaders.”

We modeled the uncivil tweets after actual tweets these politicians had written. To ensure the tweets were similarly uncivil across the target politicians, but still believable, we adapted the content of the tweet to fit with the target (e.g., attacking a Democratic leader vs. attacking a Republican leader of Congress), but otherwise kept the content identical. To create these tweets’ civil counterparts for the civil condition we edited the language, removing uncivil language and personal attacks. Across conditions we kept the displayed time of each tweet, the number of likes, retweets, and comments constant.

**Stimuli**.

Uncivil and civil tweets by President Trump and Senator Sanders that participants were randomly assigned to read.


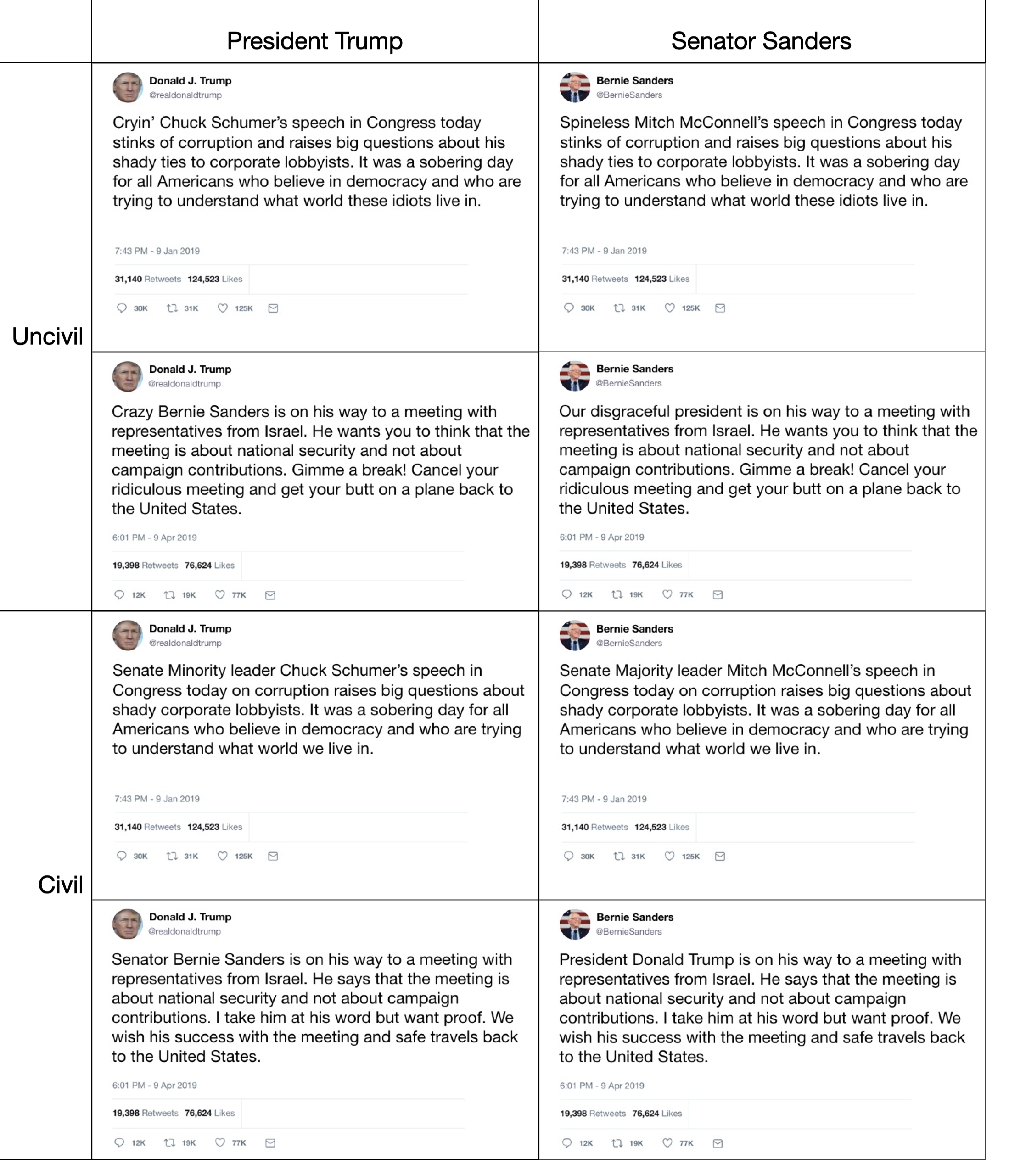


***Interest***. The question asked, “How interested are you in reading more tweets by President Donald Trump [Senator Bernie Sanders]?” Responses were on a 101-point scale anchored at 0 (*not at all*), 33 (*slightly*), 67 (*somewhat*), and 100 (*very*).

***Participant’s party***. The question asked, “Which political party do you prefer?” with response options being “Republican Party” and “Democratic Party”. We also included a question tapping participants’ level of partisanship by asking, “How much do you prefer this party over the other one?” with response options being 1 (*slightly*), 2 (*moderately*), and 3 (*extremely*); Analyses included an assessment of whether controlling for levels of partisanship yield similar results as dichotomizing participants politically.

***Twitter use***. As people who are on Twitter are not representative of the population we also measured whether participants were on Twitter or not, asking “Are you on Twitter?” with response options being “yes” (*n* = 293) and “no” (*n* = 213). We used this measure as a covariate in all analyses.

***Manipulation check***. The civility manipulation check question asked, “How civil were the tweets that you read?” with response options being -3 (*very uncivil*), -2 (*uncivil*), -1 (*slightly uncivil*), 0 (*neutral*), 1 (*slightly civil*), 2 (*civil*), and 3 (*very civil*).

**Results**

**Manipulation check**. The manipulations were successful. Participants judged the civil tweets to be more civil (*M* = 1.21, *SD* = 1.52) than the uncivil tweets (*M* = -1.24, *SD* = 1.65), *t*(527) = 17.74, *p* < .001, *d* = 1.55.

**Effect of incivility on follower interest**. To test the effect of incivility on follower interest, while also examining the potential moderating influence of the target politician and participant party affiliation, we conducted a 2(incivility vs. civility condition) x 2(Republican vs. Democratic politician) x 2(Republican vs. Democratic participant) between-subjects ANCOVA (controlling for being on Twitter), predicting interest in reading more tweets from the target politician. The analysis yielded an unqualified main effect of incivility (see Table below): interest levels were lower after reading the uncivil tweets (*M* = 38.92, *SD* = 36.27) than after reading civil tweets (*M* = 46.43, *SD* = 34.33).

**Supplementary Table 9**. Full factorial results looking at the effects of incivility manipulation, participant’s political party, and target politician’s political party, while also controlling for participant’s Twitter use. (Pilot study)

| **Predictor** | **F value** | **P value** | **Effect Size** |
| --- | --- | --- | --- |
| On Twitter | *F*(1, 493) = 19.28 | *p* < .001 | *d* = .40 |
| Incivility Manipulation | *F*(1, 493) = 8.10 | *p* = .005 | *d* = .26 |
| Participant’s Party | *F*(1, 493) = .11.00 | *p* < .001 | *d* = .30 |
| Politician’s Party | *F*(1, 493) = 11.04 | *p* < .001 | *d* = .30 |
| Incivility x Participant | *F*(1, 493) = .58 | *p* = .448 | *d* = .06 |
| Incivility x Politician | *F*(1, 493) = 1.34 | *p* = .249 | *d* = .11 |
| Participant x Politician | *F*(1, 493) = 96.11 | *p* < .001 | *d* = .88 |
| Incivility x Participant x Politician | *F*(1, 493) = .098 | *p* = .754 | *d* = .03 |

**2-way interactions looking at “party match vs. mismatch” x Incivility Manipulation**

We used the same procedure as in Study 2 described above to examine the possible influence of co-partisanship. Results of this analysis are reported below in Supplementary Table 10. As above, these analyses involved creating a new variable that indicates whether the participant and the target politician were from the same party (coded as 1) or from different parties (coded as 0).

**Supplementary Table 10.** Examination of the 2-way interactions looking at “party match vs. mismatch” x Incivility Manipulation. (Pilot Study)

| **Predictor** | **F value** | **P value** | **Effect Size** |
| --- | --- | --- | --- |
| Incivility Manipulation | *F*(1, 497) = 7.63 | *p* = .006 | *d* = .25 |
| Political Party Match | *F*(1, 497) = 114.48 | *p* < .001 | *d* = .96 |
| Incivility x Party Match | *F*(1, 497) = .76 | *p* = .384 | *d* = .09 |

**Supplementary Study 2**

**Effects of Incivility in the Media**

In this study, we asked participants to review a left-wing media video headline and a right-wing media video headline, side-by-side, and indicate which they would prefer to watch. Both headlines were from real social media (YouTube) channels and both were uncivil. We created civil counterparts by editing the text and systemically varied whether the left- or right-wing headline was uncivil (or both). This design allowed us to test whether incivility in social media headlines draw or deflect attention.

**Method**

**Participants**. We recruited 1006 participants from Amazon’s Mechanical Turk. Participants were 39 years old on average (*SD* = 12). The sample was 54% male and 46% female. The study was conducted in late November 2020, shortly after the major media networks declared Joe Biden the victor of the 2020 U.S. presidential election. Party affiliation was as follows: Democrat (*n* = 546), Independent (*Neither/Both Equally; n =*201), and Republican (*n* = 258).

**Procedure.** Participants reviewed pairings of news headlines with each pairing including a headline from MSNBC, a liberal news network, and a headline from Fox News, a conservative news network, and then selected the one they preferred. The original headlines were real (from YouTube) and uncivil. We created an MSNBC-is-civil condition by altering the wording to be more civil while leaving the Fox News headline uncivil. And we created a Fox-News-is-Civil condition by altering the wording of the Fox News headline while leaving the MSNBC headline uncivil. Participants were randomly assigned to review six pairings that all conformed to one of the three conditions (both are uncivil, MSNBC is civil, or Fox News is civil) and indicate their selection. To assess potential downstream effects of media incivility, we asked about party preference, likelihood of voting, and enthusiasm for voting in the next Presidential election. Finally, participants completed manipulation checks and reported demographics.

***Media selection and headline incivility manipulation*.** The six pairings of headlines in their civil and uncivil forms are shown below. The instructions were, “if you were going to watch a political news video right now, which would you choose to watch?” Participants then selected one or the other headline. The original headlines were real and relatively uncivilly worded headlines from YouTube. Each headline appeared with the original thumbnail from YouTube, which included a Fox News channel or MSNBC insignia. For the civility conditions, we revised the language of the headline to be more civil. There were thus three conditions, determined by random assignment: (a) the control condition had two uncivil headlines, (b) the MSNBC-is-Civil condition displayed the uncivil Fox News headline and a civil MSNBC headline, and (c) the Fox-News-is-Civil condition displayed uncivil MSNBC headlines and civil Fox News headlines. In three of the six pairings, the Fox News headline appeared above the MSNBC headline and vice versa in the remaining three. The pairings were presented in random order for each participant.

**Stimuli.**

| Pairing | MSNBC | | Fox News | |
| --- | --- | --- | --- | --- |
|  | Uncivil (Original) | Civil | Uncivil (Original) | Civil |
| 1 | President Donald Trump, the Cheater-in-Chief? | President Donald Trump, the Commander-in-Chief? | Hannity: Joe Biden facing backlash for being creepy | Hannity: Joe Biden facing backlash |
| 2 | Julián Castro: Donald Trump Administration ‘Sloppiest’ On Security Matters | Julián Castro: Trump Administration’s Record On Security Matters | Loony Left update: Democrats vote ‘present’ on the Green New Deal | Democrats vote ‘present’ on the Green New Deal |
| 3 | Chuck Rosenberg on Bill Barr: ‘What I See is Incredibly Repugnant’ | Chuck Rosenberg on Bill Barr: ‘What I See is Incredible’ | Hannity calls Mark Cuban’s politics ‘nuts’ in heated interview | Hannity challenges Mark Cuban’s politics in heated interview |
| 4 | ‘Magically Protected’: Why Hardcore Trump Supporters Won’t Wear Masks at Rally | Why Some Trump Supporters Won’t Wear Masks at Rally | Brian Kilmeade slams efforts to destroy history, tear down America’s monuments | Brian Kilmeade questions efforts to destroy history, tear down America’s monuments |
| 5 | Maxine Waters: President Donald Trump is ‘The Most Deplorable Person’ | Maxine Waters: President Donald Trump May Have Broken The Law | Tucker: The rise of left-wing rage mobs in America | Tucker: The rise of left-wing activism in America |
| 6 | ‘Moscow Mitch’ Under Fire For Ignoring Election Security | Senate Majority Leader McConnell Facing Questions Regarding Election Security | Sen. Graham pins Pelosi’s attack on Trump as ‘shameful, disgusting’ | Sen. Graham offers response to Speaker Pelosi’s allegations of President Trump |

***Downstream effects of incivility.*** To test whether there are downstream effects of media incivility on democratic intentions, we asked participants to also report their preferred candidate in the next presidential election, their enthusiasm for voting, and the likelihood that they will vote.

*Party voting intention*. The question was, “Who would you vote for if the 2024 U.S. Presidential Election were held today?” Reponses were on a 201-point scale anchored at -100 (*definitely Democrat*), -50 (*probably Democrat*), 0 (*undecided*), 50 (*probably Republican*), and 100 (*definitely Republican*).

*Voter enthusiasm*. The question was, “How enthusiastic are you about voting in the 2024 U.S. Presidential Election?” Responses were on a 101-point scale anchored at 0 (*not at all*), 25 (*slightly*), 50 (*moderately*), 75 (*very*), and 100 (*extremely*).

*Vote likelihood*. The question was, “How likely is it that you will vote in the 2024 U.S. Presidential Election?” Reponses were on a 201-point scale anchored at 0 (*not at all*), 25 (*slightly*), 50 (*moderately*), 75 (*very*), and 100 (*extremely*).

***Manipulation checks***. To check the manipulation on the Fox News headline manipulation, a question asked, “How civil were the Fox News headlines that you saw earlier, overall?” with responses being on a 201-point scale anchored at -100 (*extremely uncivil*), -50 (*uncivil*), 0 (*neutral*), 50 (*civil*), and 100 (*extremely civil*). A similar question was asked about the MSNBC headlines to check the manipulation on the MSNBC language.

***Party identification***. Participants indicated their general party preference by responding to the question, “What party do you prefer?”

**Results**

**Manipulation checks**. The manipulations were successful. Participants judged the original, uncivil Fox News headlines to be more uncivil, *M* = -25, *SD* = 50, than the headlines that were altered to be less uncivil, *M* = -5, *SD* = 54, *t*(977) = 5.68, *p* < .001, *d* = 0.38. Likewise, participants judged the original uncivil MSNBC headlines to be more uncivil, *M* = -5, *SD* = 50, than the headlines that were altered to be less uncivil, *M* = 13, *SD* = 47, *t*(1000) = 5.47, *p* < .001, *d* = 0.37.

**Effect of civility on interest and engagement**. Incivility decreased news headline selections for people across the political spectrum but had no detectable downstream effects on electoral intentions. A 3 (Headline Incivility: Both are uncivil, Fox News is civil, MSNBC is civil) × 3 (Party: Democrat, Independent, Republican) between-subjects ANOVA predicting the number of times participants made Fox News selections in the forced choice paradigm yielded a large main effect of party (see Table below), with Republicans choosing Fox News 4.58 out of 6 opportunities (*SD* = 1.67), Independents choosing Fox News 2.86 times (*SD* = 1.89), and Democrats choosing Fox only 1.17 times (*SD* = 1.45; see Figure below).

**Supplementary Figure 7**. The number times participants made Fox News selections out of six forced choice pairings wherein there was one Fox News and one MSNBC headline from which to choose. Participants were randomly assigned to review uncivil headlines from both networks or conditions in which either the MSNBC or Fox News headline was revised to be more civil. Error bars are 95% confidence intervals. (Supplementary Study 2)

The omnibus also yielded a main effect, albeit small, of Headline Incivility but no interaction, meaning that we found an effect of incivility on selections that did not differ by party. To decompose the omnibus and assess the effect of each manipulation, we conducted 2 (Headline Incivility) × 3 (Party) ANOVAs, dropping the interaction term in the models given that the interaction did not approach significance in the omnibus (see Table 4). Comparing conditions in which Fox News was civil and MSNBC was uncivil versus the opposite (Fox was uncivil and MSNBC was civil), we found that the more uncivil headline received fewer selections, suggesting that incivility is a losing strategy in an attention economy. This incivility disadvantage was attributed to both a loss of selections from MSNBC’s incivility and to a Fox News losing selections due to its incivility (see Table 4). The effect of incivility headlines had a limited reach, not influencing party preference, voter enthusiasm, or the likelihood of voting in the next presidential election (see Table below).

**Supplementary Table 11**. Tests of whether headline incivility altered, and participant party predicted, news headline selection. Tests were 3 (Headline Incivility) × 3 (Party) between-subjects ANOVAs. (Supplementary Study 2)

|  |  |  | **Dependent Variable** | | | | | | | | | | | | |
| --- | --- | --- | --- | --- | --- | --- | --- | --- | --- | --- | --- | --- | --- | --- | --- |
|  |  |  | **News Selections** | | | **Party Voting Intention** | | | | **Voter Enthusiasm** | | | **Vote Likelihood** | | |
|  |  | **Predictor** | ***F*** | ***p*** | **η_p_^2^** | ***F*** | ***p*** | **η_p_^2^** | ***F*** | | ***p*** | **η_p_^2^** | ***F*** | ***p*** | **η_p_^2^** |
| **Omnibus** | | |  |  |  |  |  |  |  | |  |  |  |  |  |
|  |  | Headline Civility | 9.33 | <.001 | .018 | 0.71 | .490 | .001 | 1.20 | | .301 | .002 | 0.10 | .903 | <.001 |
|  |  | Party | 421.99 | <.001 | .459 | 1539.13 | <.001 | .756 | 37.61 | | <.001 | .070 | 22.77 | <.001 | .044 |
|  |  | Civility x Party | 1.14 | .336 | .005 | 0.54 | .705 | .002 | 0.90 | | .461 | .004 | 0.43 | .787 | .002 |
| **Decompositions** | | | ***F*** | ***p*** | **η_p_^2^** |  |  |  |  | |  |  |  |  |  |
|  | MSNBC vs. Control | | |  |  |  |  |  |  | |  |  |  |  |  |
|  |  | Headline Civility | 4.11 | .043 | .006 |  |  |  |  | |  |  |  |  |  |
|  |  | Party | 325.11 | <.001 | .495 |  |  |  |  | |  |  |  |  |  |
|  | Fox News vs. Control | | |  |  |  |  |  |  | |  |  |  |  |  |
|  |  | Headline Civility | 7.70 | .006 | .011 |  |  |  |  | |  |  |  |  |  |
|  |  | Party | 280.68 | <.001 | .457 |  |  |  |  | |  |  |  |  |  |
|  | Fox News vs. MSNBC | | |  |  |  |  |  |  | |  |  |  |  |  |
|  |  | Headline Civility | 20.60 | <.001 | .030 |  |  |  |  | |  |  |  |  |  |
|  |  | Party | 247.58 | <.001 | .426 |  |  |  |  | |  |  |  |  |  |

**Discussion**

This study found that incivility in media headlines lowered behavioral intentions to view the associated content and did so across the political spectrum. The interest-depressing effect of incivility generalized to both left-leaning media (MSNBC) and to right-leaning media (Fox News) but was limited to the associated content and did not have downstream effects on interest to vote, party preference, or voter enthusiasm.

1. Given the relationship between power and effect size is often non-linear at small effect sizes, this study may have been underpowered. [↑](#footnote-ref-1)
